# A distinct subpopulation of membrane vesicles in *Pseudomonas putida* is enriched in enzymes for lignin catabolism

**DOI:** 10.1128/aem.01617-25

**Published:** 2025-10-01

**Authors:** Allison Z. Werner, Richard J. Giannone, Matthew J. Keller, Christine Plavchak, Dana L. Carper, Paul E. Abraham, Rebecca A. Wilkes, Ludmilla Aristilde, Davinia Salvachúa, S. Kim Ratanathanawongs Williams, Robert L. Hettich, Gregg T. Beckham

**Affiliations:** 1 Renewable Resources and Enabling Sciences Center, National Renewable Energy Laboratory 53405, Golden, Colorado, USA; 2 Center for Bioenergy Innovation, Oak Ridge National Laboratory 6146, Oak Ridge, Tennessee, USA; 3 Biosciences Division, Oak Ridge National Laboratory 6146, Oak Ridge, Tennessee, USA; 4 The Bredesen Center for Interdisciplinary Research and Graduate Education, University of Tennessee, Knoxville, Tennessee, USA; 5 Department of Chemistry, Colorado School of Mines 3557, Golden, Colorado, USA; 6 Department of Civil and Environmental Engineering, McCormick School of Engineering and Applied Science, Northwestern University 3270, Evanston, Illinois, USA; Kyoto University, Kyoto, Japan

**Keywords:** outer membrane vesicles, extracellular vesicles, biological lignin valorization, proteomics, lipidomics

## Abstract

Bacterial membrane vesicles (MVs) mediate diverse microbial processes and are emerging as powerful biomedical tools, but MV population heterogeneity remains an open question. Here, we separate, enumerate, and characterize two MV populations from the soil bacterium *Pseudomonas putida* during growth with or without lignin-derived carbon, a major carbon source from plant cells in the rhizosphere. Small MVs (MV-S, diameter ~100 nm) were produced from all cultures, whereas large MVs (MV-L, diameter ~300 nm) were observed during the late stationary phase of lignin cultivations. MV-S contained selectively packaged proteins with diverse physiological functions, whereas the MV-L proteome was smaller and largely enriched in outer membrane proteins. Interestingly, enzymes known to mediate the catabolism of lignin-derived aromatic compounds were enriched in MV-S. Overall, this study highlights the need for careful consideration of MV populations in microbial systems.

**IMPORTANCE:**

Membrane vesicles (MVs) are extracellular lipid bodies that can be generated by single-cell microbes and contain biological cargo. Since their discovery, MVs have been shown to exhibit multiple functions, including nutrient acquisition, pathogenesis, and signaling. In soil, the breakdown of plants releases aromatic compounds from lignin, and it has been previously shown that a model bacterium that consumes aromatic compounds forms MVs with enzymes responsible for the consumption of aromatic compounds. Intriguingly, a small population and a large population of MVs were observed, and it was not known if they served the same or different functions. Here, MVs isolated from bacterial growth experiments on lignin were fractionated and characterized, revealing that distinct MV populations have distinct cargo and, thus, distinct functions.

## INTRODUCTION

Membrane vesicle (MV) production by bacteria is a conserved phenomenon, with implications in diverse biological roles of biomedical and environmental relevance (1–3). A wide range of human immune responses are reportedly mediated by bacterial MVs, including cardiac dysfunction (4), inflammatory responses (5), and transmission of HIV (6). Accordingly, engineered MVs are being developed as vaccine and drug delivery platforms (6, 7). More recently, MVs have been shown to play roles in marine nutrient cycling (3), degradation of synthetic polymers (8), and catabolism of lignin-derived aromatic compounds (9, 10). However, the environmental implications of MVs are not well-characterized and remain an area of active investigation.

Many knowledge gaps remain around MV cargo packaging, biogenesis, and cell-to-cell interactions (1, 5). The small size of MVs poses technical and logistical challenges for research. However, the application of super-resolution microscopy has facilitated spatiotemporal studies (11) and provided evidence of direct delivery of DNA by MVs to epithelial cells (12). In 2018, Zhang et al. reported that mammalian extracellular vesicles are heterogeneous, and different-sized populations contained distinct signaling pathways (13, 14). This discovery advanced understanding of mammalian MVs, but research into bacterial MV systems has largely lagged despite exciting evidence of bacterial MV subpopulations (15).

**Fig 1:**
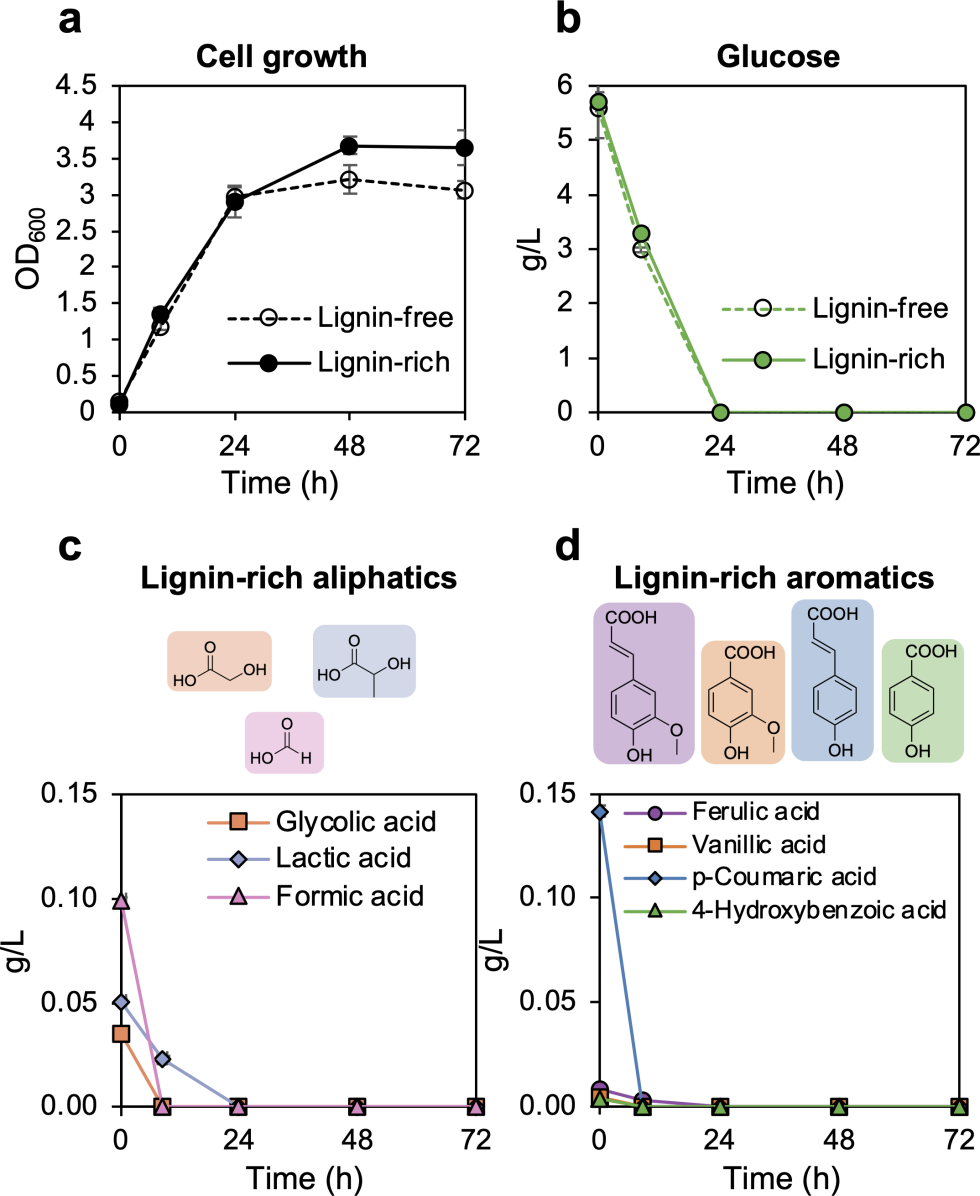
Growth and substrate utilization by *P. putida* during lignin-rich and lignin-free cultivations. (**a**) Growth and (**b**) glucose utilization during cultivation on lignin-rich and lignin-free media with samples collected at 24 and 72 h for total MV (MV_T_) isolation by an affinity column. (**c**) Aliphatic and (**d**) aromatic substrate utilization in the lignin-rich cultivations. All experiments were conducted in biological triplicate; average ± standard deviation is plotted. *p*HBA, *p-*hydroxybenzoic acid; VA, vanillic acid; *p*CA, *p*-coumaric acid; and FA, ferulic acid.

Bacterial MV heterogeneity is well recognized as a barrier to understanding composition, biogenesis, and function (5). In 2020, Salvachúa and coauthors reported that gram-negative saprotrophic soil bacterium *Pseudomonas putida* KT2440 secretes MVs that are selectively packaged with enzymes active in the catabolism of lignocellulose-derived aromatic compounds (Fig. 1a) (10). Two sizes of MVs were observed during aromatic catabolism, but whether they were functionally or compositionally distinct was not determined. *P. putida* has been extensively engineered for the conversion of lignin-derived aromatics into biochemicals (16), making an understanding of aromatic catabolism in this species critical for both understanding carbon cycling and advancing the bioeconomy (17).

Here, we determined that the two populations of MVs produced by *P. putida* are compositionally distinct during growth on lignin-rich media through a combination of analytical separations (18), bottom-up proteomics, lipidomics, and quantitative proteomics. MV population enumeration showed that the larger-sized MV population is produced in lignin-rich but not lignin-free conditions. Comparative analyses showed that the small and large MV populations contain different proteomes, with MV-S selectively packaged with diverse catabolic machinery, including aromatic catabolic enzymes (19). However, <1% of the total protein pool for the aromatic catabolic enzymes was found in the MV fraction. Overall, this work establishes a methodological platform for the separation and study of MV heterogeneity in bacterial systems, proposes a model for the secretion of distinct MV populations, and improves our understanding of aromatic catabolism in the well-studied biochemical production chassis *P. putida*.

## RESULTS

### Asymmetric flow field-flow fractionation with online multi-angle light scattering revealed that *P. putida* secretes two MV subpopulations based on size

*P. putida* was cultivated in media with glucose plus lignin liquor from alkaline pretreated corn stover (“lignin-rich”) or glucose only (“lignin-free”) as a control. The alkaline pretreated corn stover was provided at 25% (vol/vol), and thus, the relative concentrations of aromatic and aliphatic compounds are proportionally representative of the feedstock and pretreatment (20–23). These compounds include glycolic, lactic, and formic acids (Fig. 1c) in addition to lignin-derived aromatics, *p*-coumarate, *p*-hydroxybenzoate, ferulate, and vanillate (Fig. 1d). Lignin-rich cultivations consumed the lignin-derived aromatic and aliphatic compounds within 24 h and grew to a higher OD_600_ compared with lignin-free conditions (*P-*value = 0.007) (Fig. 1a; Data Set S1). Glucose was depleted after 24 h in both conditions (Fig. 1b). The total MV fraction (MV_T_) was harvested from lignin-rich and lignin-free cultivations at 24 and 72 h using an affinity column, as employed in our previous study (10).

**Fig 2:**
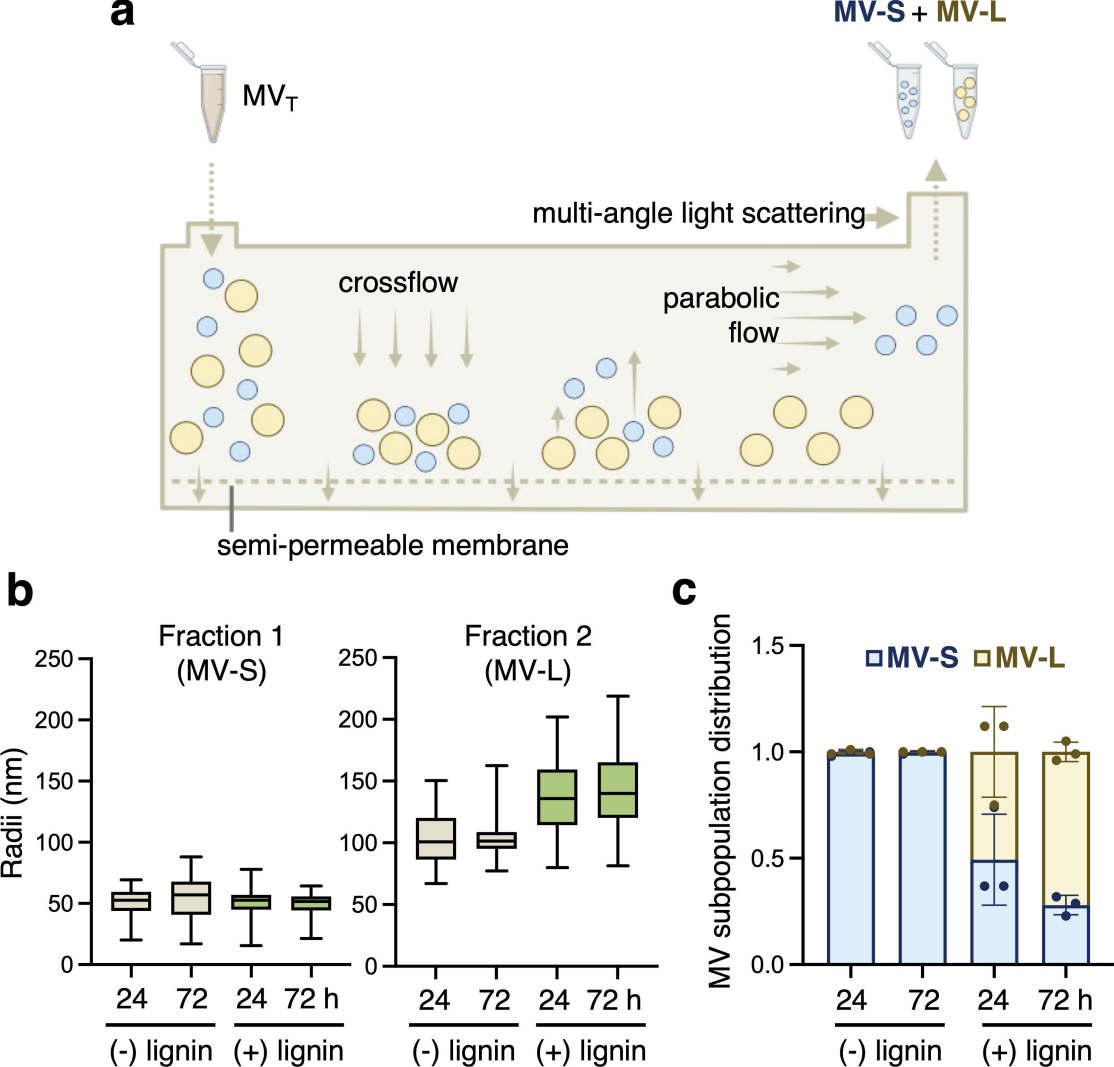
*P. putida* secretes two populations of MVs that were separated and enumerated by AF4-MALS. (**a**) Schematic of MV analysis methods. MV_T_ was fractionated into small (MV-S) and large (MV-L) fractions via AF4 and enumerated and sized via online MALS. (**b**) Particle radii as determined by MALS (refractive index of 1.5) for fraction 1 (“MV-S”) and fraction 2 (“MV-L”) in lignin-rich and lignin-free samples harvested at 24 and 72 h. Whiskers represent min to max values of all MV measurements from biological triplicates, each with a technical duplicate (**c**) MV population distribution as a percentage of the total collected MV_T_ (MV-S + MV L) in lignin-rich and -free samples harvested at 24 and 72 h. Subpopulation distributions were calculated from particle counts for each biological triplicate (each with technical duplicate measurements); each triplicate value is plotted, and error bars represent the standard deviation across biological triplicates. All experiments were conducted in biological triplicate; MALS measurements were additionally conducted with technical duplicates. Partially created with Biorender.com. All raw data provided in Data Set S1.

To characterize each MV population individually, we developed an asymmetrical flow field-flow fractionation (AF4) with an online multi-angle light scattering (MALS) workflow (18). AF4 utilizes a channel that lacks a stationary phase but contains a semi-permeable membrane that acts as an analyte accumulation wall. During separations, a perpendicular crossflow induces a field which pushes analytes to the membrane; meanwhile, translational diffusion counteracts that field, which leads to the separation of particles based on hydrodynamic diameter (Fig. 2a).

Two AF4 fractions corresponding to the previously observed size populations (10) were separated, enumerated, and sizes were determined at 24 and 72 h (Fig. 2b; Figs. S1 and S2; Tables S1 to S3). Fraction 1 consisted of an MV population with an average radius of 51 ± 4 nm, termed the “small MV” subpopulation (MV-S). Fraction 2 consisted of an MV population with an average radius of 121 ± 22 nm, termed the “large MV” subpopulation (MV-L). MV-S consisted of >99% of the total MV population in lignin-free conditions; conversely, MV-S accounted for only 59% and 29% at 24 and 72 h, respectively, of the total MV population in lignin-rich conditions (Fig. 2c; Fig. S2).

The MV-S population was observed in both media conditions and time points, and the concentration did not change between 24 h and 72 h. Although the average diameter of MV-S was consistent across time and media condition, the average MV-L diameter was 49 ± 6 nm larger (*P* = 0.002) in lignin-rich compared with lignin-free conditions at both 24 h and 72 h (*p*_24 h_ = 0.003; *p*_72 h_ = 0.004). Furthermore, the lignin-rich cultivations exhibited a 68% increase in MV-L secretion from 24 to 72 h (*P* = 0.003) despite the sizes remaining the same (274 ± 20 and 288 ± 16 nm, respectively). These data show an induction of the larger but not the smaller MV population in lignin-rich conditions.

### Bottom-up proteomics analysis revealed that the two MV subpopulations have distinct proteomes

**Fig 3:**
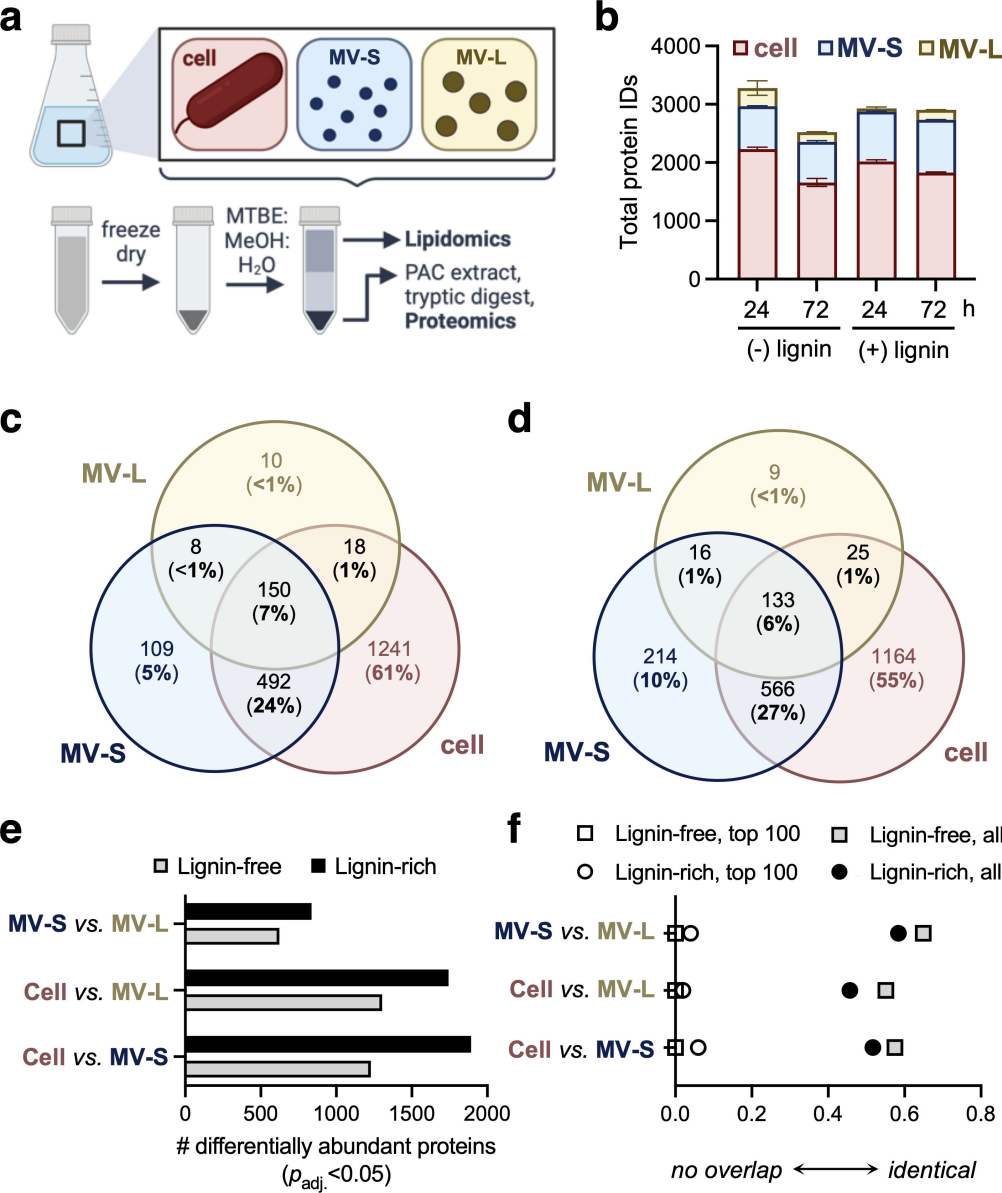
MV-S and MV-L have distinct protein cargo. (**a**) Schematic of multi-omic preparation from cell, MV-S, and MV-L fractions within a given cultivation. MTBE, methyl tertiary butyl ether; MeOH, methanol; PAC, protein aggregation capture. (**b**) Total number of identified proteins in each fraction. Error bars represent standard deviation across biological triplicates. (**c**) Venn diagram of binary presence/absence analysis of protein identifications across each fraction at 72 h in (**c**) lignin-free and (**d**) lignin-rich conditions. (**e**) Number of differentially abundant (*p*-adjusted (*p*_adj_.) <0.05) proteins between fractions at 72 h. (**f**) Rank-based overlap (rbo) values considering all proteins or the top 100 most abundant proteins identified in each fraction at 72 h. All analyses were performed on data from three biological replicates. (**a**) Created with Biorender.com.

We next sought to understand the similarities and differences between the MV-S and MV-L proteomes using bottom-up proteomics. Protein and lipid content of the cell pellet and each MV population from a single culture were recovered via a biphasic extraction on lyophilized samples corresponding to the fractions and particle counts described above (Fig. 3a). Across all conditions and time points, a rich proteome was observed in the MV-S population, with an average of 799 ± 90 protein identifications from biological triplicates; conversely, MV-L contained considerably fewer total protein identifications (175 ± 111) (Fig. 3b; Fig. S3; Excel 1). Principal component (PCA) and non-metric multidimensional scaling (NMDS) analyses indicated that overall data variation was driven by media composition and cultivation time (Figs. S4 and S5). MV-L samples at 24 h had few protein identifications and poor PCA clustering within biological replicates; hence, these were omitted from differential protein abundance analyses.

Binary presence/absence analyses were first conducted to assess the overall distribution of the proteome across the three compartments (Fig. 3c and d; Figs. S6 and S7). At 72 h, when MV-L abundance was maximal in lignin-rich cultivations, 55% of the identified proteins were found exclusively in the cell, 10% were found exclusively in the MV-S, and <1% were found exclusively in the MV-L. The MV-S population in lignin-rich conditions contained approximately twice the number of protein identifications compared with lignin-free conditions. Only considering proteins in the MV_T_ fraction, 20% and 15% of the proteins were identified in both MV-S and MV-L in lignin-free and lignin-rich cultivations, respectively (Fig. S7).

Differential abundance analyses were conducted across media conditions and fractions (Figs. S8 and S9; Excel 1). Both MV-S and MV-L had more differentially abundant proteins compared with the cell in lignin-rich and lignin-free conditions (Fig. 3e). When comparing between MV populations at 72 h, 94% (783 proteins) and 97% (602 proteins) of the differentially abundant proteins had increased abundance in the MV-S compared with MV-L. In lignin-rich conditions, MV-L had 54 enriched proteins, primarily with MetaCyc spatial classifications of the outer membrane or MetaCyc functional classifications related to regulation (Fig. S10). Conversely, the enriched MV-S proteome had diverse MetaCyc functional classifications, including biosynthesis, degradation, energy, transcription/translation, and regulatory roles (Fig. S10) (24).

As MV fractions contained 6%–50% of the total protein identifications made in the cell pellet, and the MV-L contained 6%–43% of the protein identifications made in the MV-S, we considered whether differential abundance analyses may not accurately capture enrichment due to different sample complexities. To assess this possibility, rank-order analysis was used to compare the similarity of fractions based on the order of protein abundance within each fraction, which can mitigate the effect of the total number of proteins. Rank-based overlap (RBO) analysis was conducted on the top 100 proteins from each fraction and all proteins in a given fraction (Fig. 3f; Table S4). RBO values provide a measure of similarity, with an RBO of 1 indicating identical lists and an RBO of 0 indicating no overlap. When considering only the top 100 proteins in each fraction, all three compartments were highly dissimilar regardless of media type. Considering all proteins, a much stronger overlap was observed, with less similarity observed in the lignin-rich conditions. These results, although largely in alignment with differential abundance analyses, indicate that even the MV-L is highly dissimilar from the cell when considering only the 100 most abundant proteins in each compartment.

### Quantitative proteomics confirmed that MV-S, but not MV-L, is enriched in aromatic catabolic enzymes

**Fig 4:**
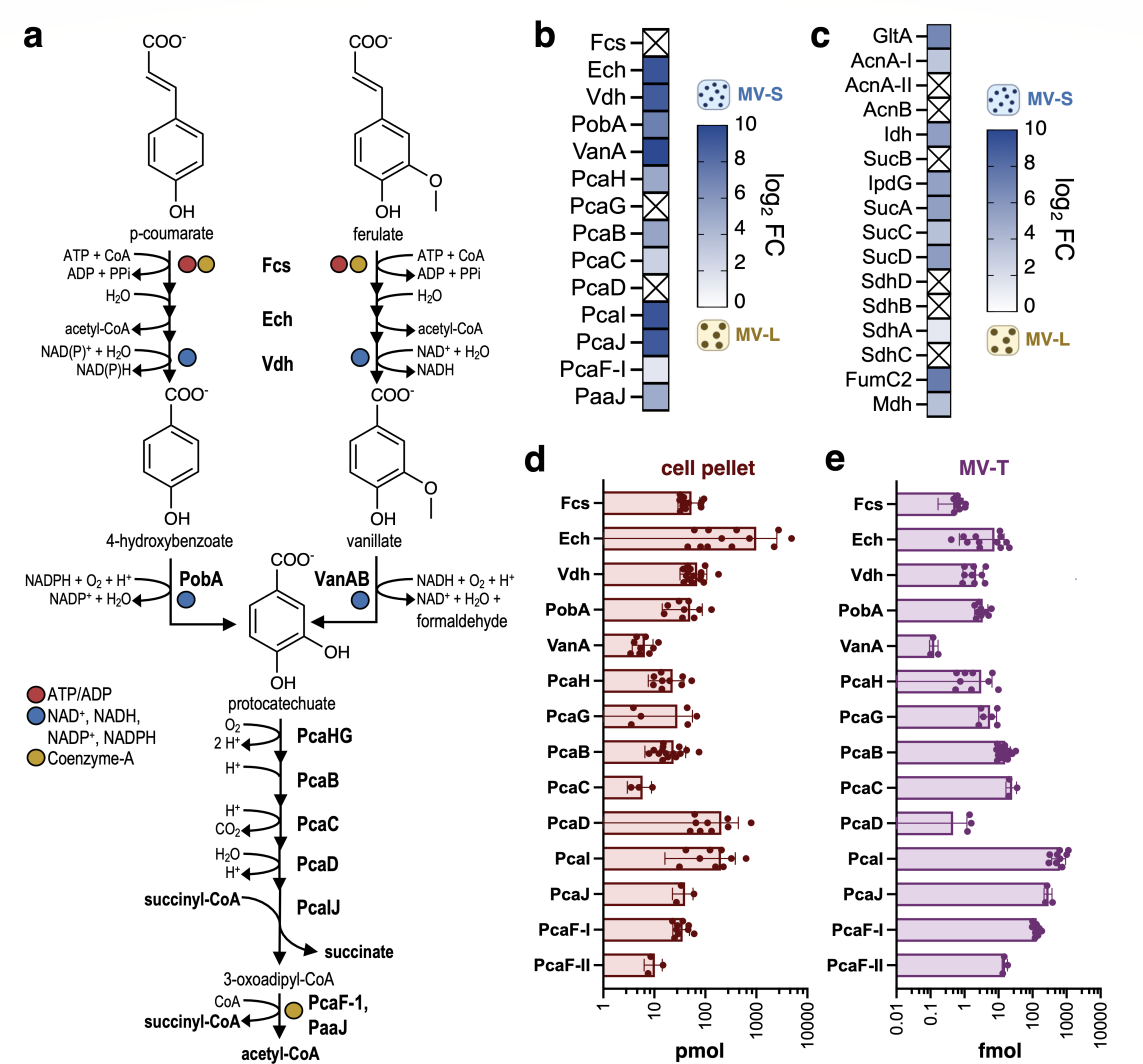
Aromatic-catabolic enzymes are enriched in MV-S compared with MV-L, but the cell harbors > 99% of the total protein pool. (**a**) Catabolic pathway for lignin-derived aromatic compounds in the β-ketoadipate pathway and entry into the TCA cycle. R = H (*p*-coumarate and *p*-hydroxybenzoate) or R = OMe (ferulate, vanillate). The heatmap showing log_2_ fold change (FC) for significantly enriched (*p*_adj_ <0.05) enzymes in the (**b**) β-ketoadipate pathway and (**c**) TCA cycle in MV-S vs. MV-L using median-centered normalization. “X” indicates *p*_adj_. >0.05; hence, fold change is not plotted. No negative log_2_ FC values were present; hence, heatmaps are shown from 0 to 10. Quantitative proteomic data for the (**d**) cell pellet fraction (in pmol) and the (**e**) MV_T_ fraction (in fmol). The distribution of each protein between the cell and MV_T_ as a percentage of the total quantified pool (“% MV”) is plotted. Individual points are shown for each labeled peptide measurement for each of the biological triplicates; error bars represent standard deviation across all measurements.

The metabolic pathway and associated biochemistries for catabolism of *p*-coumarate and ferulate—through key intermediates *p*-hydroxybenzoate and vanillate—is well understood (Fig. 4a) (25, 26). *p-*Coumarate and ferulate are first acted on by the feruloyl-CoA-synthase encoded by *fcs*; the feruloyl-CoA hydratase-lyase encoded by *ech* then performs hydration and retro-aldol reactions to create the aldehyde (*p*-hydroxybenzaldehyde and vanillin, respectively) (27). Aldehyde dehydrogenases, principally but not exclusively the vanillin dehydrogenase encoded by *vdh,* then form each respective carboxylic acid (*p*-hydroxybenzoate and vanillate) (27–29). Then, the two *p*-coumarate and ferulate degradation branches converge at protocatechuate: *p*-hydroxybenzoate by action of the *para*-hydroxybenzoate-3-hydroxylase encoded by *pobA,* and vanillate by action of the vanillate monooxygenase oxygenase encoded by *vanAB* (25, 26, 30, 31). Protocatechuate is then degraded by the *ortho*-cleavage pathway, with the first step being ring-opening by the protocatechuate 3,4-dioxigenase encoded by *pcaHG* (25, 26, 32). PcaB, PcaC, PcaD, and PcaIJ then sequentially convert the ring-opened product to tricarboxylic acid (TCA) cycle intermediates (succinate and acetyl-CoA) (26, 33, 34).

In the global proteomics data, all of the enzymes involved in the conversion of *p-*coumarate and ferulate to succinyl-CoA and acetyl-CoA were identified in the MV-S fraction from lignin-rich cultivations except for VanB (Excel 1). Of those, 11 were significantly enriched in the MV-S compared with the MV-L, regardless of the normalization scheme (Fig. 4b; Fig. S11), as were several enzymes in the TCA cycle (Fig. 4c). Conversely, with the exception of PcaF-I and PaaJ, none of the β-ketoadipate pathway enzymes were identified in the lignin-free MV fraction (Excel 1). VanB, PcaIJ, PcaF-I, and PaaJ were also considered enriched in the MV-S compared with the cell (Table S5; Fig. S12).

To determine whether this was an artifact of disparate sample complexities between the cell pellet and MV fractions, we performed quantitative proteomics on the cell pellet and MV_T_. For each protein involved in the conversion of *p*-coumarate or ferulate to TCA cycle intermediates (Fig. 4a), 2–5 isotopically labeled peptides (^13^C/^15^N on C-terminal lysine and arginine residues) were synthesized and utilized for absolute protein quantification in the MV_T_ and cell pellet via liquid chromatography and tandem mass spectrometry with parallel reaction monitoring (Fig. S13; Excel 1). Quantitation values were normalized by the volume from which proteins were extracted, such that concentrations are representative of a 50 mL culture.

As expected, based on the known gene regulation in response to aromatic compounds (26, 34), the aromatic catabolic enzymes were only observed in the lignin-rich condition (Fig. S13; Excel 1). Within the upper pathway (prior to aromatic ring-cleavage), Ech was by far the most abundant protein in the cell pellet, with over an order of magnitude higher average abundance than the related Fcs and Vdh (Fig. 4d). In MV_T_, femtomole levels of proteins were detected, again for all proteins except VanB, with the lower pathway (ring-cleavage and downstream reactions) being most abundant (Fig. 4e). Notably, the lower pathway enzymes do not require ATP or NAD(PH), whereas many of the upper pathway enzymes do. As a fraction of the total (cell plus MV_T_), the MV fraction contained less than 1% of the total protein pool.

### Lipidomics revealed that MVs have higher relative amounts of phosphatidylethanolamine than the cell, regardless of the MV size

**Fig 5:**
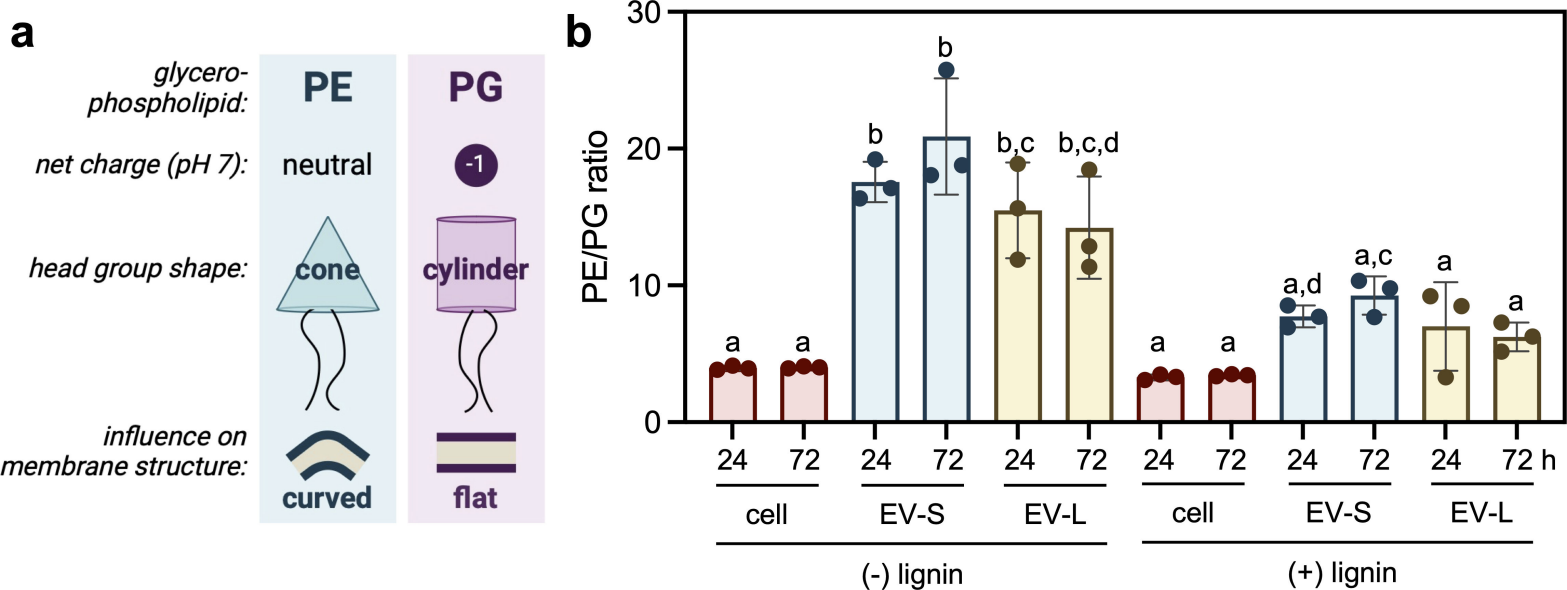
MV fractions have higher PE/PG ratios than the cell, and lignin-rich cultivations have more PG than lignin-free cultivations within each fraction. (**a**) Schematic of key differences between PG and PE glycerophospholipids. (**b**) PG ratio in the cell and MV fractions. A one-way ANOVA with Tukey’s correction for multiple comparisons was conducted to determine differences across the fractions. Different letters indicate statistically significant differences (*P* ≤ 0.05).

Finally, glycerophospholipid composition was compared across MV-S, MV-L, and the cell to evaluate any compositional differences. An untargeted lipidomics measurement was performed, from which 21 phosphatidylethanolamine (PE) and 14 phosphatidylglycerol (PG) species were identified in MV samples, and 28 PE, 29 PG, and 38 cardiolipin (CL) species were observed in cell pellet samples (Fig. 5a; Excel 1). Ceramide and hexosylceramide derived from the lignin liquor were also identified in lignin-rich MV-S samples. This observation suggests stable intercalation into the MV, as they were not removed during sample preparation. Notably, lipopolysaccharide moieties were not analyzed.

Independent of media composition, both MV populations had higher PE/PG ratios than the cell pellet (Fig. 5b; Tables S6 and S7), indicative of an increased proportion of cone-shaped PE head groups in the MV, which could impart intrinsic curvature (35) for blebbing initiation or increased stability of the MV. No difference in the PE/PG ratio was observed between the two MV populations in lignin-rich cultivations. MV-S had a notable enrichment in PG 33:1 and PE 28:1 compared with MV-L. Notably, PE/PG ratios for the cell, MV-S, and MV-L were an average of 0.6, 10.7, and 8.2 lower across both time points in lignin-rich cultivations, respectively (Fig. S14). Overall, these data suggest that although the two MV populations have similar PE/PG ratios, both contain higher PE/PG ratios than the cell.

## DISCUSSION

In this study, we showed that the proteomes of the two MV populations secreted by the gram-negative soil bacterium *P. putida* KT2440 during growth on lignin-rich media are functionally distinct. To enable this work, we developed a preparatory AF4 with an online MALS workflow for separating and enumerating the MV populations (18), and a sample preparation method to extract proteins and lipids from each population. We found that the smaller MV population, termed MV-S, accounted for >99% the total MV pool (MV_T_) in lignin-free conditions, whereas in lignin-rich conditions, it was only 29%–59% with an increase in the larger population, termed MV-L, over time. In the presence of lignin, MV-S were enriched in enzymes and proteins involved in diverse biological processes compared with MV-L, including enrichment of lignin-derived aromatic catabolic enzymes. Conversely, the MV-L population had a less rich proteome, with outer membrane proteins dominating the enriched proteome pool. Quantitative proteomics showed that the MV fraction contained <1% of the total protein pool for any given aromatic-catabolic enzyme, considering the intracellular and MV fractions. Finally, we found that the MV-S and MV-L populations both contained proportionally higher amounts of PE than the cell pellet, and that the cultures in lignin-rich media had proportionally higher amounts of PG than lignin-free media.

These results show that studying bulk MVs will confound analysis of MV function in *P. putida*. Although this study has environmental and biotechnological relevance, MV heterogeneity in other bacteria has pharmacological relevance. For example, MV size has been shown to impact uptake by epithelial cells (36) and virulence factor secretion (37). However, separating and enumerating MV populations is nontrivial. Drawing inspiration from Zhang et al. (13, 14), we adapted an AF4 method with online MALS for this purpose (18). Size-exclusion chromatography (38) and sucrose gradient (36) are additional separation methods that, when paired with methods for enumerations (e.g., MALS or nanoparticle tracking analysis), could also yield successful results.

Given the low lignin-derived aromatic catabolic enzyme loading relative to the cell, the extent to which MVs play a meaningful role in aromatic catabolism in natural environments remains an open question. Our quantitative proteomics measurements are in agreement with surface-to-volume ratio calculations that indicate MV protein capacity is limited (5). As it relates to the role of MVs in aromatic catabolism during cultivation in shake flasks, we recently investigated the effect of increased vesiculation on aromatic catabolism in *P. putida* (39). We recently observed a hypervesiculation mechanism-specific response: deletion of *oprI*, encoding a major outer membrane lipoprotein, had faster vanillate utilization, whereas deletion of *oprF,* encoding a porin F, was much more sensitive to aromatics and had reduced fitness overall (39). Currently, no genetic mutations are known that prevent MV production entirely, limiting mechanistic studies; nonetheless, we observed unaffected aromatic catabolism in a hypovesiculation mutant (*P. putida* ∆*PP_*4669) (39). In industrial bioprocess settings, MV engineering for targeted protein packaging and secretion could be employed to increase extracellular activity, if desired (e.g., for conversion of cytotoxic compounds) or as a strategy to tailor cell-free bioprocesses, which may offer advantages over microbial conversion systems in particular applications (40).

The molecular mechanism(s) of selective protein cargo packaging in bacteria remains an area of active investigation (5). The dissimilar proteomes of MV-S and MV-L could suggest divergent cargo packaging and/or biogenesis mechanisms for the two MV populations, but future mechanistic studies would be required for elucidation. In *Haemophilus influenzae* and *Vibrio cholerae*, lipid transport between the inner and outer membranes is proposed to be a natural trigger for MV blebbing (41). Notably, we recently found that modulating the homologous lipid transport system in *P. putida* did not substantially impact vesiculation (39), which does not support this mechanism in *P. putida.* Studies to track spatiotemporal MV-S and MV-L secretion dynamics would require super-resolution microscopy (11), and such an approach could additionally be used to ascertain the spatiotemporal fate of MVs in the environment.

The decreased PE/PG ratio observed in lignin-rich conditions could result in an increased relative negative charge of the membrane, in alignment with reports of bacterial phospholipid modulation to change membrane physical, structural, and mechanical properties (35). Aromatic carboxylic acids, such as ferulic and *p-*coumaric acid, interact strongly with zwitterionic lipids via electrostatic interactions, and these interactions are decreased with increasing phospholipid bilayer negative charge (42). Future investigation into the modulation of PE/PG as an adaptive response to lignin-containing media is needed to mechanistically understand the impact of lipid changes on vesiculation and lignin-rich growth.

## MATERIALS AND METHODS

### Bacterial cultivation

Wild-type *P. putida* KT2440 (ATCC 47054) was used for all experiments. *P. putida* was revived from glycerol stocks overnight in 50 mL of Miller’s LB (Sigma #L3522) in 250 mL baffled flasks at 30°C and 250 rpm. For growth experiments, *P. putida* was otherwise cultivated in M9 minimal medium with trace metals (6.78 g/L Na_2_HPO_4_, 3 g/L KH_2_PO_4_, 0.5 g/L NaCl, 1 g/L NH_4_Cl, 2 mM MgSO_4_, 100 mM CaCl_2_, 100 mM MnSO_4_, 100 mM CuSO_4_, 100 mM FeSO_4_, and 50 mM ZnSO_4_). *P. putida* was inoculated at an OD_600_ of 0.1 from the LB seed cultures into non-baffled 250 mL Erlenmeyer flasks with 50 mL of M9 minimal medium, as described above, supplemented with 5 g/L glucose alone for lignin-free or 5 g/L glucose plus 25% (vol/vol) alkaline lignin liquor from corn stover, prepared as previously described (10). Six replicates per condition were conducted. At 24 h of cultivation, the entire 50 mL of three replicates per condition was harvested for analysis; at 72 h, the entire 50 mL of the remaining three replicates per condition was harvested for analysis.

### Fractionation (cell and MV_T_)

To fractionate the cultivations into cells (cell pellet) and total membrane vesicles (MV_T_), the harvested 50 mL of culture was centrifuged for 20 min at 8,000 × *g* and 4°C. The cell pellet was frozen for later analysis. The supernatant was transferred to a new sterile 50 mL conical tube and centrifuged again for 20 min at 8,000 g and 4°C. The resulting supernatant was vacuum-filtered through a 0.45 µm pore size unit and subsequently a 0.2 µm pore size unit (ThermoFisher #596-4520). MV_T_ was then enriched from clarified samples using the ExoBacteria OMV Isolation Kit (SBI #EXOBAC100A-1), following the manufacturer’s instructions. MV_T_ samples were further processed by asymmetric flow field-flow fractionation.

### Asymmetrical flow field-flow fractionation (AF4) and multi-angle light scattering (MALS)

MV-S were fractionated into small (MV-S) and large (MV-L) populations by AF4 as recently described (18). All experiments were performed using an AF2000 system coupled to a multi-angle light scattering (MALS) DAWN HELEOS II (Wyatt Technology Corporation, Santa Barbara, California). A spacer with a nominal thickness of 350 µm, a breadth of the channel inlet of 2 cm, a breadth of the channel outlet of 0.5 cm, and a tip-to-outlet length of 27.5 cm was used to form the channel. A 30 kDa molecular weight cutoff regenerated cellulose membrane (Postnova Analytics, Salt Lake City, Utah) was used as the accumulation wall. The carrier fluid was 1× phosphate-buffered saline (PBS) and was filtered between the HPLC pump and AF4 channel using a 0.1 µm filter (Merck Millipore Ltd, Ireland). The focusing time was 15 min, the injection flow rate was 0.2 mL/min, the detector flow rate was 0.5 mL/min, and the sample injection volume was 1 mL. The crossflow rate was programmed to start at 1.0 mL/min during focusing, then decreased linearly to 0.1 mL/min over 10 min, held at 0.1 mL/min for 2.5 min, and then turned off. Fractions containing both small and large vesicles were collected and stored at −80°C until further analyses. Data acquisition and particle counting analysis were performed using Astra 7.3.2.21 (Wyatt Technology Corporation, Santa Barbara, California). The MALS detector was normalized using bovine serum albumin (BSA) (Sigma-Aldrich, St. Louis, Missouri). Particle counts for each MV population were determined using the fraction collection time intervals, and the signal intensity was heavily despiked. The particle count for each MV population was evaluated using the sphere model and a refractive index (RI) of 1.5. Particle counts and radius information for each biological replicate consisted of data from two technical replicates.

### Protein and lipid extraction

Samples were lyophilized before extraction. Lipids and proteins were extracted from the dried sample with a biphasic extraction as follows: 200 µL water, 350 µL methanol, 750 µL methyl *tert-*butyl ether (MTBE), and 3 µL EquiSplash LIPIDOMIX Quantitative Mass Spec internal standard (Avanti) were added to each sample in 50 mL conical tubes. The samples were vortexed for 20–30 s, transferred to 2 mL tubes, and sonicated in a bath on ice for >15 min. Samples were again vortexed for 30 s, incubated on an orbital shaker on ice for 1–1.5 h, and centrifuged for 10 min at 12,000 × *g* to generate solvent layers. The upper layer was transferred to a fresh tube and processed for lipid analysis. Notably, cell pellet samples only had one phase, and the entire solvent supernatant was processed for lipid analysis. For MV samples, which had biphasic separation, the middle layer was removed. In all cases, the insoluble pellet was processed for protein analysis. All samples were stored at −80°C until further analysis.

### Lipid preparation

Samples were dried under nitrogen gas and re-extracted to further remove salts and extract lipids. The same solvent ratio was used as before: 125 µL water, 150 µL methanol, and 500 µL MTBE were added to each sample, vortexed for 10 s, sonicated for 30 min, incubated on an orbital shaker on ice for 1 h, and centrifuged for 10 min at 12,000 × *g*. The upper layer was transferred to a glass vial and dried under nitrogen gas.

### LC-MS/MS analysis of phospholipids

The MV samples were resuspended in 42 µL of the 8Bu lipid solvent (8% butanol, 23% isopropanol, and 69% water), whereas the cell pellet samples were dissolved in 200 µL of 8Bu. This solvent allows for good dissolution of a variety of lipids while not interfering with the chromatography (43). A nano-LC column was created in-lab by packing a 100 µm inner diameter capillary (with a tip laser pulled) with 1.7 µm C18 stationary phase to a length of about 12.5 cm. The lipid extracts were analyzed with a reversed-phase LC-MS/MS method. This employed a 60:40 ACN:water mobile phase A and a 90:10 IPA:ACN mobile phase B, both with ~0.42 g/L ammonium acetate. The gradient started at 1% B and was held for 3 min. Over the next 1.5 min, B was increased linearly to 30%. B was then ramped more slowly to 35% over 5 min, before increasing to 55% over 6 min. Over the next 35 min, B was increased to 70%. B was then increased to 99% in 10 min, followed by a hold for 17 min. This was then decreased to the starting conditions (1% B) over 2 min, and the column was equilibrated for 9.5 min. The mass spectrometer was operated in a top 10 DDA method where one full scan was acquired at 30 k resolution with the Orbitrap, followed by 10 data-dependent CID fragmentation scans in the ion trap at 30 normalized collision energy. The spray voltage was adjusted empirically for the best electrospray performance. This was run in both positive and negative modes for all samples.

### Data analysis of lipidomics data

Lipids were identified based on a manual analysis of the data using El-MAVEN (44). Mass to charge values of PE and PG lipids with carbons ranging from 20 to 40 and degrees of unsaturation ranging from 0 to 2 were entered into El-MAVEN to generate extracted ion chromatograms (EICs). EICs were inspected for a distinct chromatographic peak. If present, any MS (2) scans associated with that peak were interrogated for fragment ions consistent with the putative lipid. Lipids fragment in a predictable manner, thus allowing fragment ions to be generated in a rule-based manner. Lipids matching the exact mass and MS (2) patterns were considered identified, and their peak areas were integrated. Although in some cases, an unambiguous fatty acid tail assignment could be made, in most cases, the EICs consisted of two or more overlapping lipids with differing fatty acids with the same total carbons/unsaturation. Thus, lipids were condensed into total carbon species, and any subspecies (if present) were summed together. Lipids of the same class with the same unsaturation degree will elute with a strongly linear mass-to-charge vs retention time relationship. This was also used in some cases to confirm lipid identifications. In the case of cardiolipin, which contains four different fatty acids, all species with between 60 and 80 carbon atoms and up to five degrees of unsaturation were manually investigated. This analysis resulted in 38 Cl species being identified in the cell pellets.

### Protein preparation

Insoluble pellets from cell pellet samples were resuspended in 1 mL of 4% SDS-UB. To remove salts from the insoluble pellet in AF4-MV insoluble pellet samples and lignin-free VFS insoluble pellet samples, the insoluble pellet was resuspended in 750 µL of water, vortexed, and incubated at 37°C to solubilize salts. The resulting solution was passed through a Vivaspin 500 10 K MWCO filter (Sartorius, #VS0102) to retain protein while removing salts in solution. After passing through the sample, the filter was washed twice in 100 mM Tris (UB buffer), 200 µL 4% SDS in UB was added to the filter, incubated >15 min at RT, and transferred to a new tube. To remove salts from these samples, 4 mL of water was added, the samples were vortexed, and heated to 37°C to achieve salt solubilization. The resulting suspension was centrifuged at 4,000 × *g* for 15 min to pellet the lignin while maintaining proteins in solution. The resulting suspension (supernatant) was passed through a Vivaspin 2 500 10K MWCO filter (Sartorius, #VS0202) to retain proteins on the filter while removing the salts in solution. Again, proteins were removed by incubation with 4% SDS-UB on the filter and transferring to a fresh tube.

To the collected proteins in 4% SDS-UB, 10 mM DTT was added, pulse vortexed, incubated for 10 min at 90°C, pulse vortexed, and centrifuged for 10 min at 21,000 × *g*, 30 mM IAA was added, and incubated for 15 min in the dark. Next, 4 µL of magnetic beads (Sera-Mag Magnetic Beads Carboxyl Hydrophilic, Wilmington, DE) were added and mixed by gently pipetting. Then, 3 volumes of acetonitrile were added, vortexed for 3 s, incubated at RT for 10 min, gently vortexed, and incubated at RT for an additional 10 min. Tubes were then placed on a magnetic rack for 2 min to collect the beads. Beads were washed in acetonitrile and 70% ethanol in water before resuspension in 100 µL fresh ABC. Proteins were digested with 0.5 mg of Pierce™ MS Grade Trypsin (ThermoSci #90058) at 37°C overnight, and an additional 0.5 mg of trypsin was added and incubated at 37°C for 3–5 h. Samples were then placed on the magnetic rack for >2 min, the supernatant was filtered through a Vivaspin 500 10 K MWCO filter (Sartorius, #VS0102), and the flow-through was dried by speedvac. Samples were resuspended in 25 µL of Solvent A (2% (vol/vol) acetonitrile plus 0.1% formic acid in water) and stored at −20°C prior to analysis.

### Non-targeted LC-MS/MS analysis of proteins

*P. putida* cells were collected by centrifugation, while MVs residing in the supernatant were isolated and fractionated by size using asymmetric flow field-flow fractionation (AF4). All fractions were freeze-dried, resuspended in ammonium bicarbonate buffer (pH 8), and filtered/desalted (10 kDa MWCO; Vivaspin PES spin column (Sartorius). Cell pellets were lysed through a combination of bead-beating, detergent (adjusted to 4% SDS), and heat treatment, followed by protein reduction (dithiothreitol), cysteine blocking (iodoacetamide), and isolation using protein aggregation capture (PAC) and trypsin proteolysis (45, 46). MV and supernatant fractions were adjusted to 4% SDS, and proteins were isolated and digested similarly. Tryptic peptides derived from the processed samples were acidified, filtered, and injected onto a trap column in-line with an in-house pulled nanospray emitter (47). Peptides were analyzed via data-dependent acquisition (DDA) liquid chromatography-tandem mass spectrometry (LC-MS/MS) over a 120 min organic gradient (47). Resulting MS/MS spectra were searched against the *P. putida* KT440 proteome database as detailed below.

### Targeted quantitative analysis of aromatic catabolic proteins

Stable isotope-labeled (SIL) peptides corresponding to proteins of the beta-ketoadipate pathway were procured, mixed, and initially analyzed by both DDA and targeted parallel reaction monitoring (PRM) LC-MS/MS. This enabled the determination of the dominant charge state, retention time, and fragmentation spectrum of each specific SIL peptide. Both 25 and 50 fmol loading amounts were interrogated. From these analyses, a refined list of proteotypic peptides, along with their dominant product ions, was prepared using Skyline software (v.23.1.0.455) (48). Cell pellets and fractionated/enriched MVs were isolated and processed for LC-MS/MS analysis using methods previously described (10). For MV samples, a modified chloroform-methanol extraction (CME) method (49) was employed due to the absence of a visible interfacial protein “pancake.” Here, the top aqueous layer was discarded, and neat methanol was added, followed by centrifugation at 21,000 × *g* to precipitate interfacial proteins. The recovered MV proteins were denatured, reduced, digested with trypsin, acidified, filtered, and freeze-dried. Dried, endogenous MV peptides were resuspended in 40 µL of solvent A2 (2% acetonitrile in 98% LC-MS-grade water with 0.1% formic acid) and SIL peptides were spiked into the resuspended sample such that 50 fmol of each SIL peptide was present per 25% of the total sample. Twenty-five percent of each sample was then injected onto an in-house pulled nanospray emitter (75 µm ID, packed to 15 cm with 1.7 µm C18 resin [Kinetex, Phenomenex]) and analyzed using PRM LC-MS/MS with retention time scheduling (determined above). For cell pellet-derived peptides, a similar preparation and PRM analysis was performed, but rather SIL peptides were spiked at a ratio of 50 fmol per 1500 ng of endogenous peptides injected. Thus, each cell pellet PRM run represented 0.050%–0.083% of the total processed cell pellet. PRM LC-MS/MS analysis was conducted over a 120 min organic gradient, measuring both heavy (SIL) and light (endogenous) versions of each proteotypic peptide. PRM acquisition parameters include a resolution of 17,500, AGC target of 2e5, isolation window of 1.8 m/z with a 0.3 m/z offset, and applying a normalized collision energy (NCE) of 27. Skyline software was used for PRM data processing to quantify peak areas and calculate the ratios between spiked-in SIL peptides (50 fmol) and their corresponding endogenous peptides, therefore enabling precise quantitation of proteins within the beta-ketoadipate pathway.

### Data analysis of proteomics data

All MS raw data files were analyzed using the Proteome Discoverer software (Thermo-Fisher Scientific, version 2.1) (50). Each MS raw data file was processed by the SEQUEST HT database search algorithm (51), and confidence in peptide-to-spectrum (PSM) matching was evaluated by Percolator (52). Peptides and PSMs were considered identified at q < 0.01, and proteins were required to have at least one unique peptide sequence. Proteins with at least one unique peptide were exported from Proteome Discoverer. Log2-transformation of protein abundances was performed, followed by local regression (LOESS) normalization and mean-centering across the entire data set in R using scripts from the InfernoRDN software (v1.1.7995) (53). The abundance values for proteins with missing values were imputed with random values drawn from the normal distribution (width 0.3, downshift 2.2) using R. Lignin-rich fraction 2 samples at 24 were not used for this analysis because of so few proteins being identified. Normalized imputed data were used to generate an NMDS plot and perform a PERMANOVA across relevant variables using the vegan package (v2.6-2) in R. Rank-biased overlap (RBO) was carried out using the gespeR package (v.1.26.0) in R using non-imputed data. RBO was used to get a measure of similarity between lists of proteins; output values range between 0 and 1, where 0 means no similarity and 1 means identical lists. Bio-replicates were averaged to create the ranked list of proteins with no weight given to any position within the list. Two calculations for each comparison were used: (i) the top 100 proteins from each group, and (ii) all proteins were identified.

## Data Availability

Proteome, targeted PRM quantitation, and lipidome raw data and results are available through the MassIVE and ProteomeXchange repositories under accessions MSV000096959 and PXD060204, respectively. Data files are directly located at ftp://massive.ucsd.edu/v09/MSV000096959/. All other data are provided in Data Set S1.

## SUPPLEMENTAL MATERIAL

Data Set S1 aem.01617-25-s0001.xlsx Raw data.

Supplemental material aem.01617-25-s0002.pdf Figures S1 to S14; Tables S1 to S7.

ASM does not own the copyrights to Supplemental Material that may be linked to, or accessed through, an article. The authors have granted ASM a non-exclusive, world-wide license to publish the Supplemental Material files. Please contact the corresponding author directly for reuse.
