# Supplementary material for: A distinct subpopulation of membrane vesicles in Pseudomonas putida is enriched in enzymes for lignin catabolism
Source: Appl Environ Microbiol. 2025 Oct 1;91(10):e01617-25. doi: 10.1128/aem.01617-25 (PMC12542622; doi:10.1128/aem.01617-25)
Supplement: Supplemental material — Figures S1 to S14; Tables S1 to S7. [file aem.01617-25-s0002.pdf]

Supplementary Information

A distinct subpopulation of membrane vesicles in Pseudomonas putida is enriched in enzymes for lignin catabolism

Allison Z. Werner,[1,2] Richard J. Giannone,[2,3] Matthew J. Keller,[3,4] Christine Plavchak,[5] Dana L. Carper,[2,3] Paul E. Abraham,[2,3] Rebecca A. Wilkes,[1,2] Ludmilla Aristilde,[6] Davinia Salvachúa,[1,2] S. Kim Ratanathanawongs Williams,[5] Robert L. Hettich,[2,3,*] Gregg T. Beckham[1,2,*]

[1]Renewable Resources and Enabling Sciences Center, National Renewable Energy Laboratory, Golden, CO 80401
[2]Center for Bioenergy Innovation, Oak Ridge National Laboratory, Oak Ridge, TN 37830
[3]Biosciences Division, Oak Ridge National Laboratory, Oak Ridge, TN 37831
[4]The Bredesen Center for Interdisciplinary Research and Graduate Education, University of Tennessee, Knoxville, TN 37996
[5]Department of Chemistry, Colorado School of Mines, Golden, CO 80401
[6]Department of Civil and Environmental Engineering, McCormick School of Engineering and Applied Science, Northwestern University, Evanston, IL 60208, USA

Correspondence: gregg.beckham@nrel.gov; hettichrl@ornl.gov

SUPPLEMENTAL FIGURES

Figure S1. Individual AF4-MALS fractograms. 2
Figure S2. MALS particle counts. 3
Figure S4. Total protein IDs. 4
Figure S5. Proteomic data normalization and principal component analysis (PCA) plots for (a-b) the cell pellet, (c-d) the MV-S, and (e-f) the MV-L. 5
Figure S6. Non-metric multidimensional scaling (NMDS) analysis. 7
Figure S7. Venn Diagram of protein content in all three fractions within a given cultivation. 8
Figure S8. Number of shared and unique proteins identified in MV-S vs. MV-L at 72 h. 9
Figure S9. Comparison of data normalization scheme impact on differentially abundant proteins. 10
Figure S10. Number of differentially abundant proteins in all comparisons made. 11
Figure S11. MetaCyc spatial and functional analysis of differentially-abundnat proteins. 12
Figure S11. Heatmap comparison of log_2 fold-change between MV_S and MV_L in lignin-rich media at 72 h across data centering/normalization strategy. 13
Figure S12. Heat map of differential abundance of beta-ketoadipate and TCA cycle related proteins between the MV_S and cell pellet. 14
Figure S13. Abundance of each protein as detected by 3-5 unique peptides for (a) cell pellet samples and (b) pooled MV samples for both lignin-free and lignin-rich experiments. 15
Figure S14. Phosphatidylethanolamine (PE) to phosphatidylglycerol (PG) ratios in cell pellets. 16

SUPPLEMENTAL TABLES

Table S1. AF4 collection times. 17
Table S2. Particle counts. 18
Table S3. Particle sizes. 19
Table S4. Rank-based overlap (rbo) values. 20
Table S5. Log_2 fold-changes for beta-ketoadipate pathway enzymes. 21
Table S6. Linear model analysis of untargeted lipidomics data from MVs. 22
Table S7. Linear model analysis of untargeted lipidomics data from cell pellets. 23

SUPPLEMENTAL FIGURES

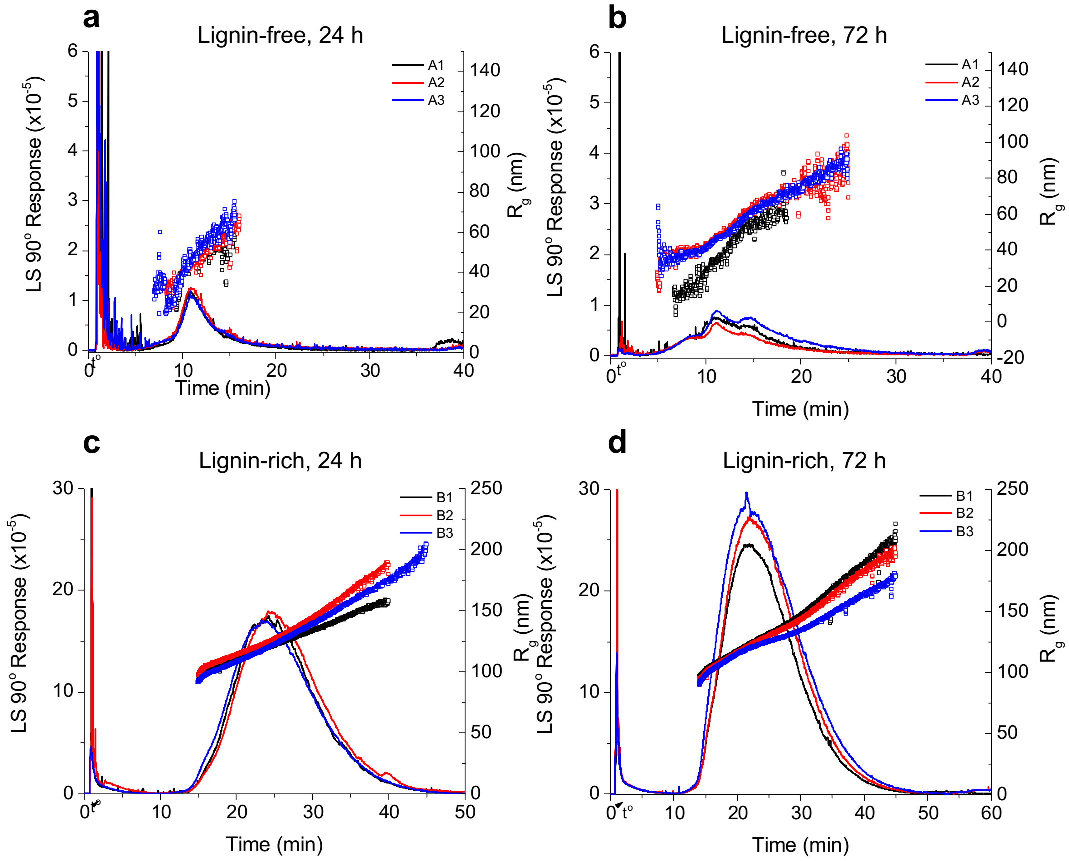

Figure S1. Individual AF4-MALS fractograms.
Lignin-free cultivations at (**a**) 24 h and (**b**) 72 h, and lignin-rich cultivations at (**c**) 24 h and (**d**) 72 h. Each biological replicate is shown as a separate trace. A1-3 denote the three biological replicates in lignin-free conditions; B1-3 denote the three biological replicates in lignin-rich conditions.

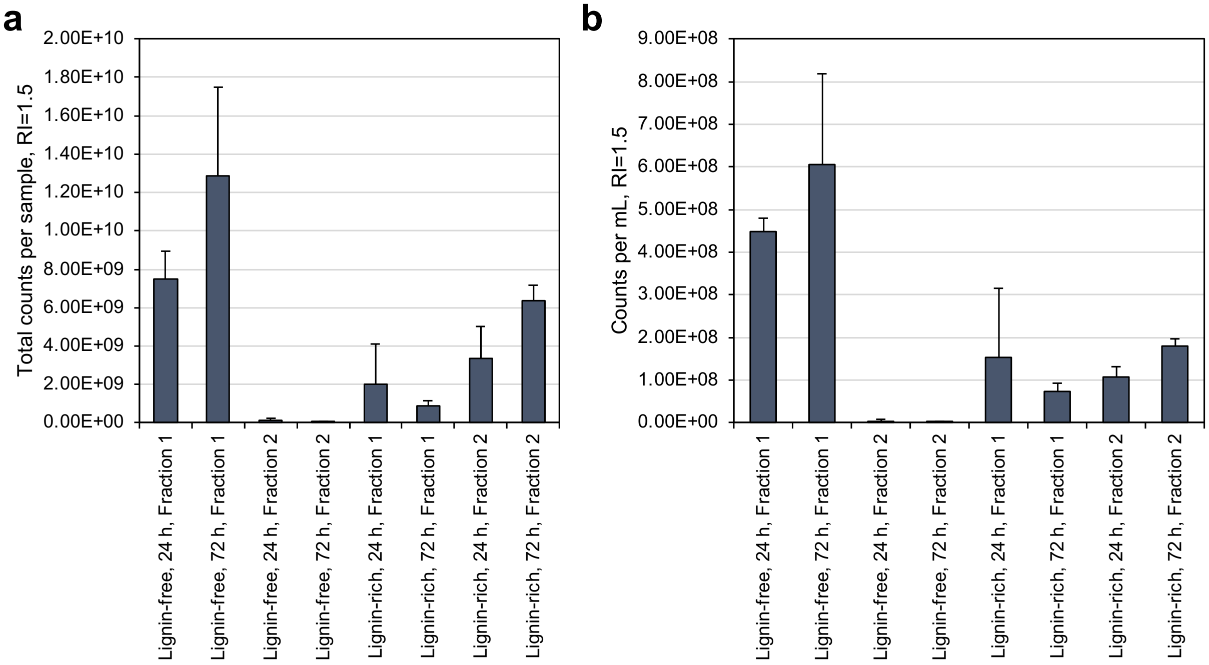

Figure S2. MALS particle counts.
(**a**) Total particle counts per sample at an RI of 1.5. (**b**) Particle counts per milliliter at an RI of 1.5. Average ± standard deviation, n=3. Fraction 1 corresponds to MV-S, and Fraction 2 corresponds to MV-L.

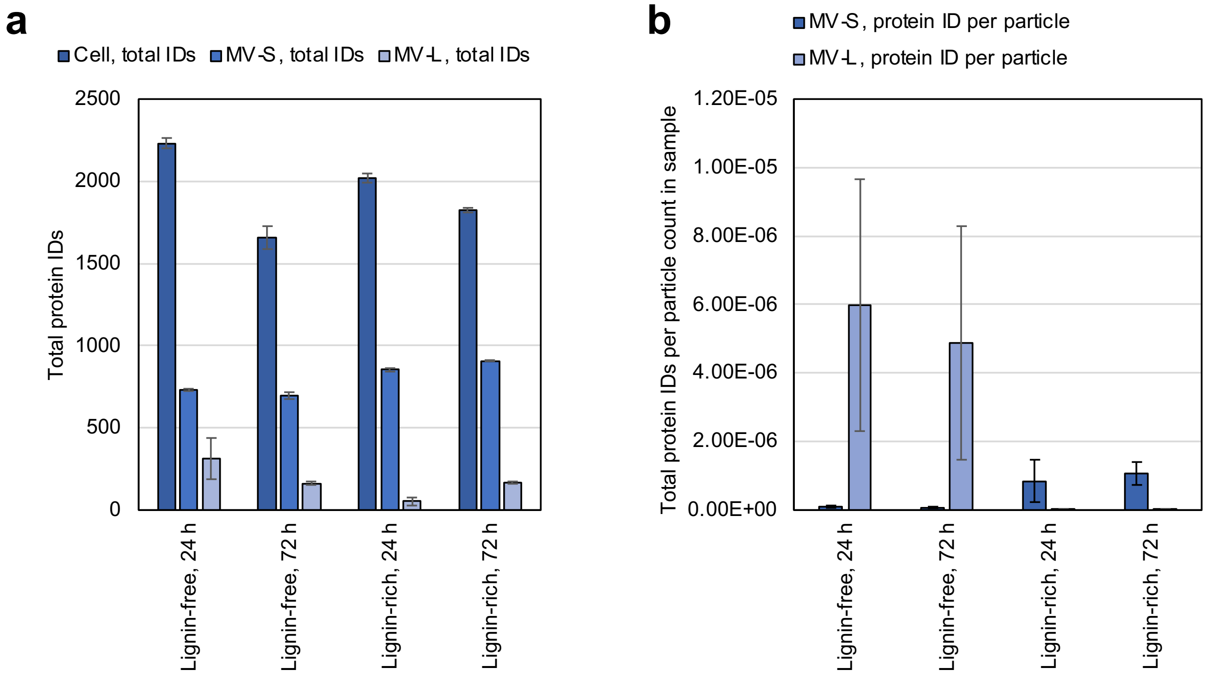

Figure S3. Total protein IDs.
(**a**) Total protein IDs in each sample. (**b**) Total protein IDs divided by particle count in MV-S and MV-L samples. Average ± standard deviation, n=3.

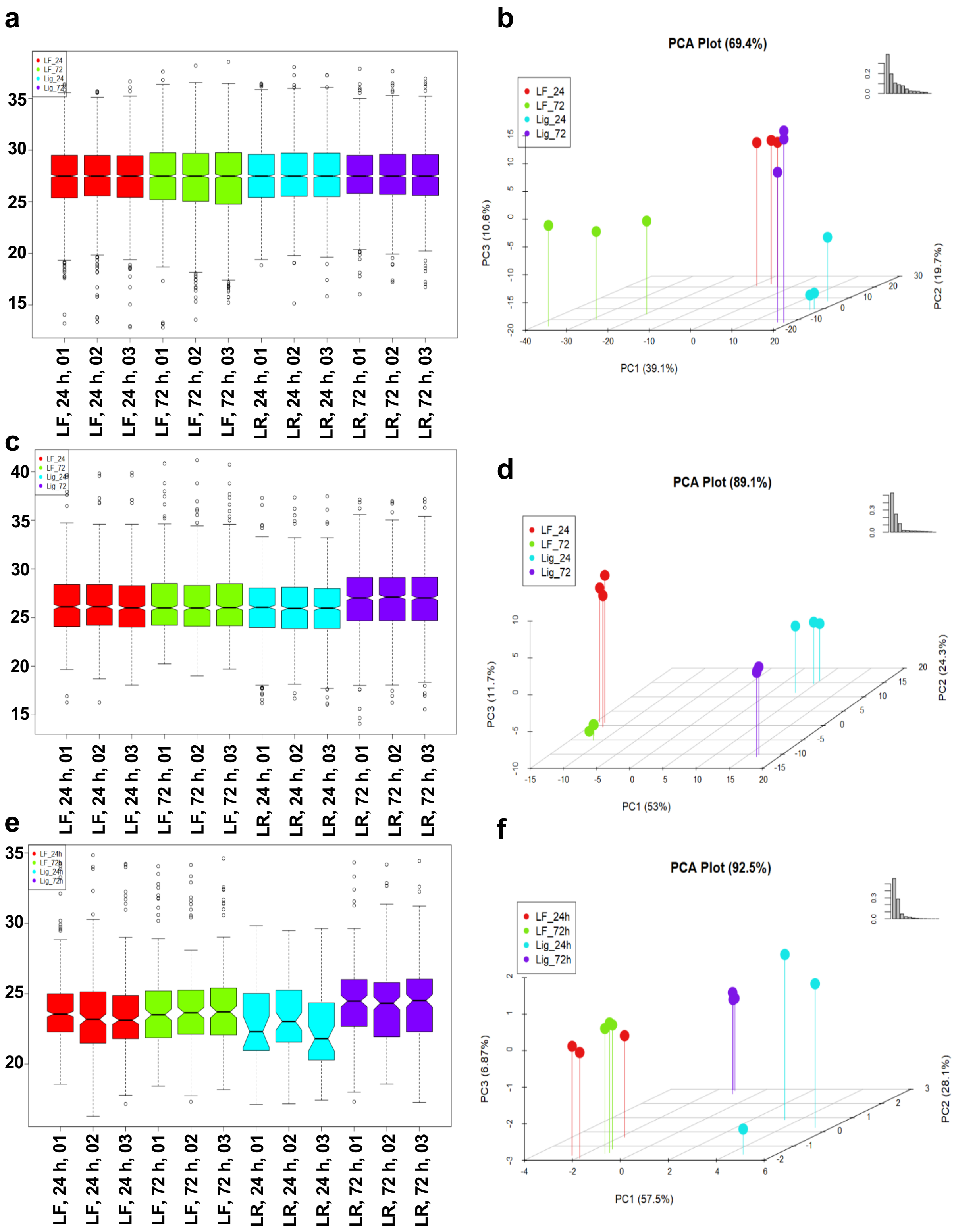

Figure S4. **Proteomic data normalization and principal component analysis (PCA) plots for (a-b) the cell pellet, (c-d) the MV-S, and (e-f) the MV-L**.
Cell pellet (**a**) LOESS normalized, median centered data and (**b**) PCA plot. MV-S (**c**) LOESS normalized, median centered data and (**d**) PCA plot. MV-L (**e**) LOESS normalized, median centered and (**f**) PCA plot.

LF = lignin-free; LR = lignin-rich. Each biological replicate is shown independently, denoted by 01, 02, and 03. The box-and-whisker plots depicted in (**a**), (**c**), and (**e**) use individual points to denote data points which fall outside the “whiskers”; we note that these are not defined as “outliers” in the statistical sense and were not removed from the datasets.

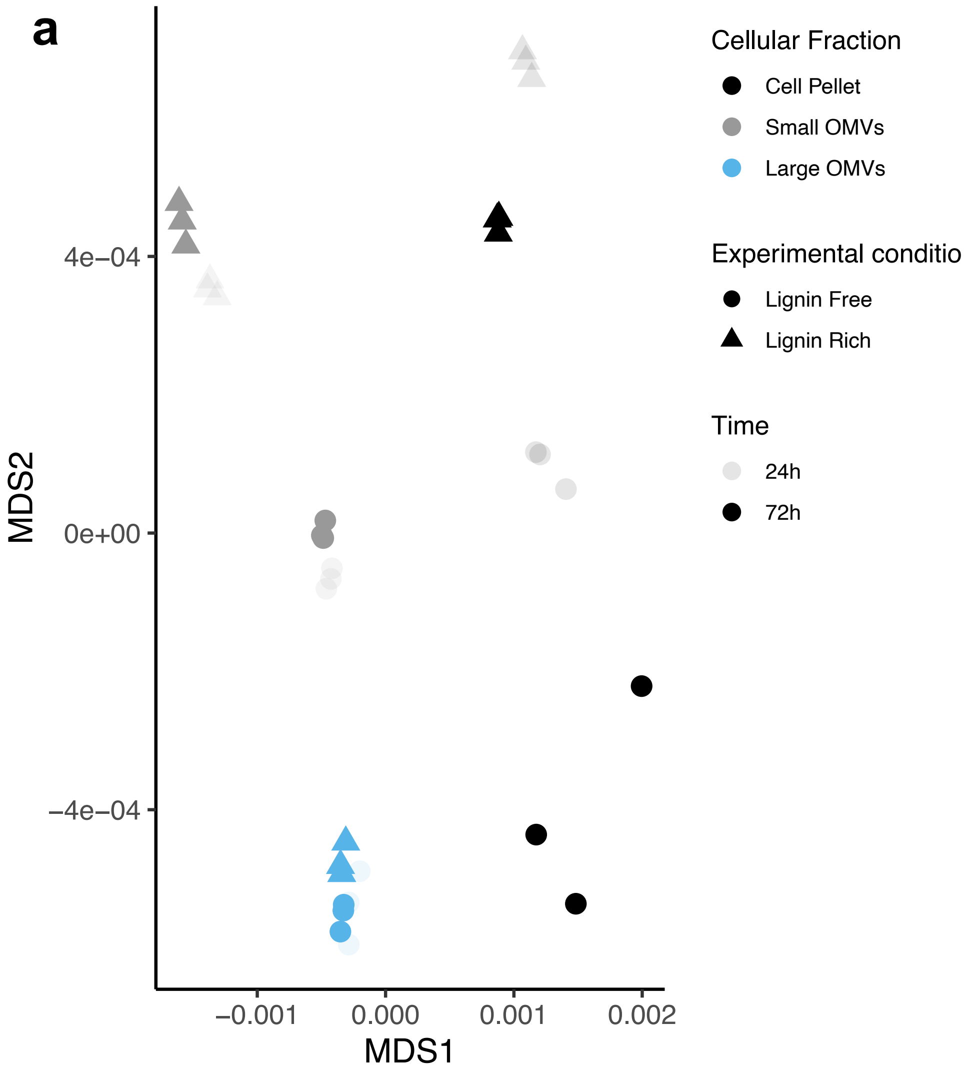

Figure S5. Non-metric multidimensional scaling (NMDS) analysis.
Euclidean distances of all samples colored by cellular fraction, with different shapes indicated lignin-rich and lignin-free cultivation conditions, and shading indicating 24 and 72 h collection time-points.

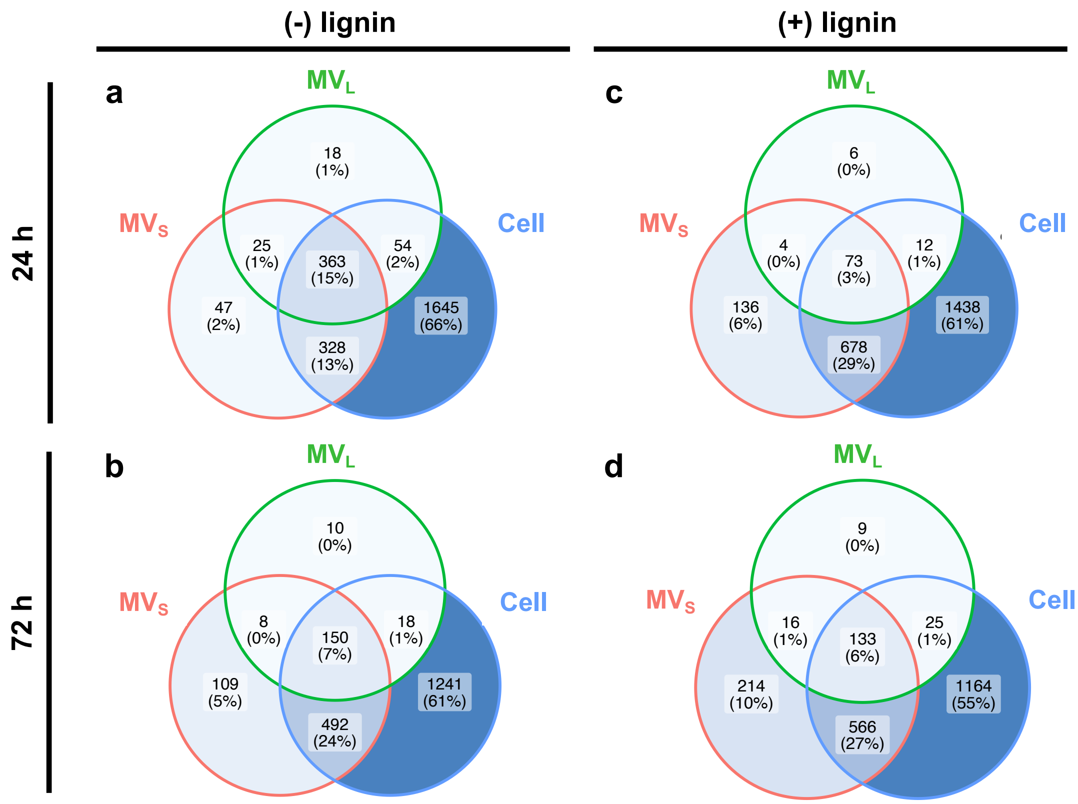

Figure S6. Venn Diagram of protein content in all three fractions within a given cultivation. Lignin-free cultivations at (**a**) 24 h and (**b**) 72 h. Lignin-rich cultivations at (**c**) 24 h and (**d**) 72 h. Number of proteins and percentage as a function of total observed proteins across all samples shown is displayed.

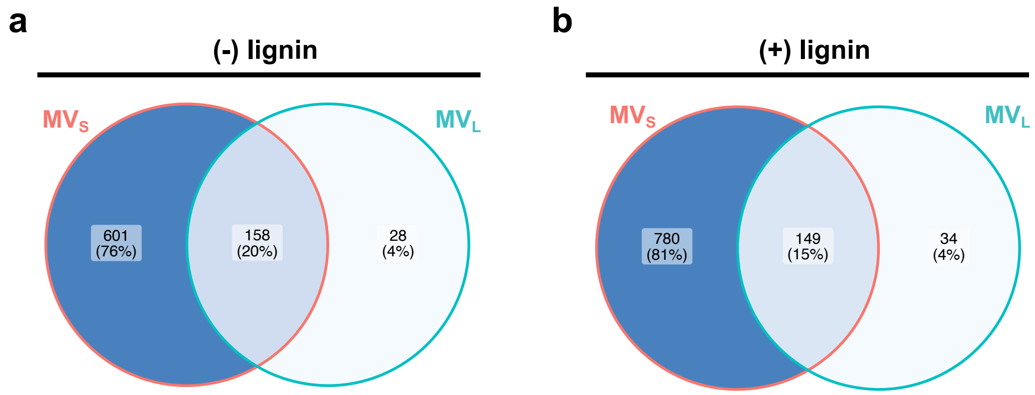

Figure S7. Number of shared and unique proteins identified in MV-S vs. MV-L at 72 h.
(**a**) Lignin-free 72 h comparison. (**b**) Lignin-rich 7 h comparison. Percentages represent distribution across total observed proteins in the MV_S and MV_L compartments

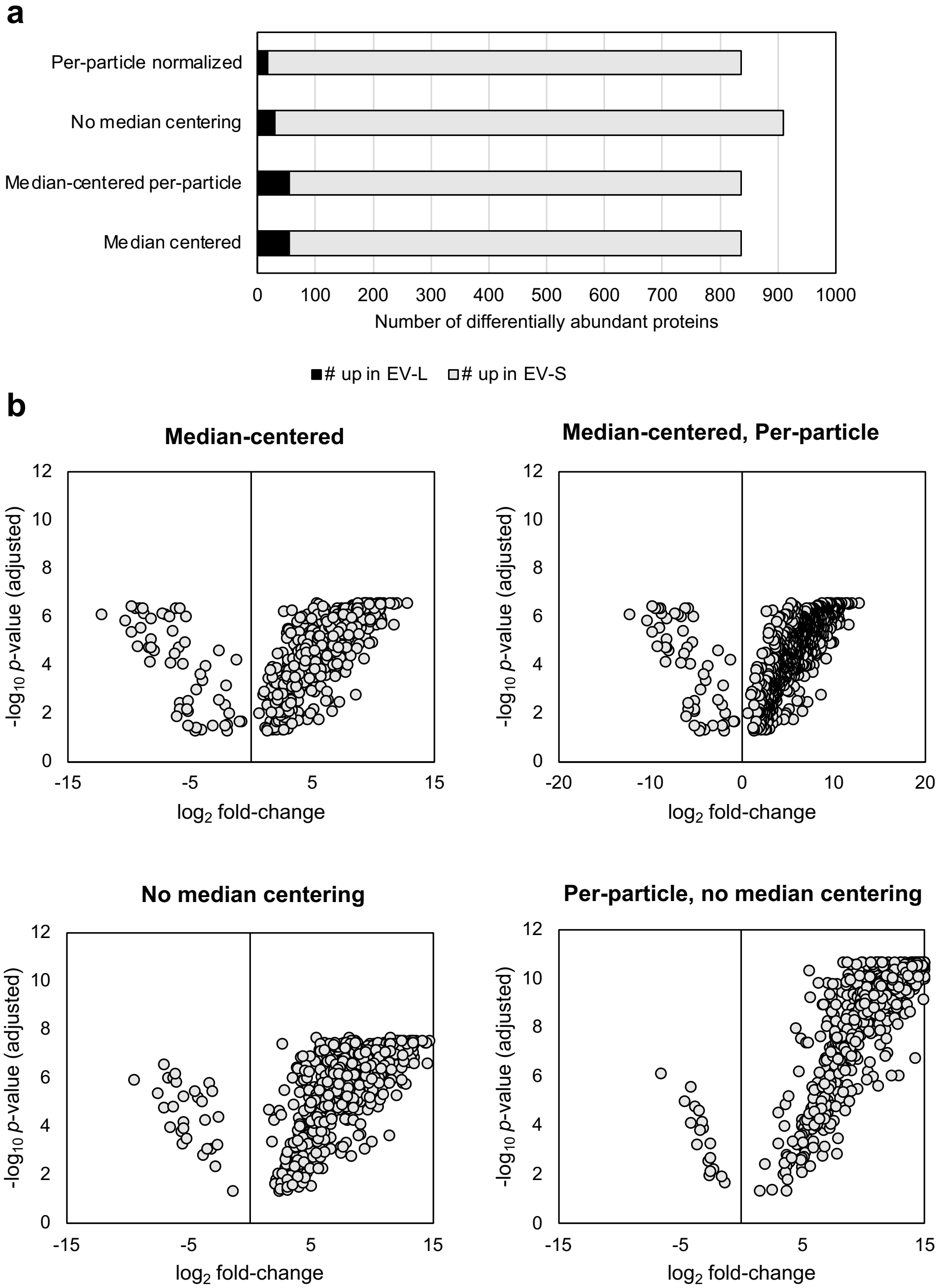

Figure S8. Comparison of data normalization scheme impact on differentially abundant proteins. (**a**) Comparison of the number of differentially abundant proteins 72 h in lignin-rich cultivations across four different data processing/normalization schemes. From Limma output filtered datasets. (**b**) Volcano plots of differentially abundant proteins in a comparison of MV_S and MV_L at when the protein abundance data is median-centered, median-centered and per-particle normalized, not centered or normalized, and per-particle normalized but not median-centered. The total number of statistically significant differentially abundant proteins between the MV_S and MV_L increased slightly without median-centering, the number of proteins deemed significantly increased in the MV_L was higher in analyses with median-centering, and particle normalization did not affect the number proteins in each category within median-centered data.

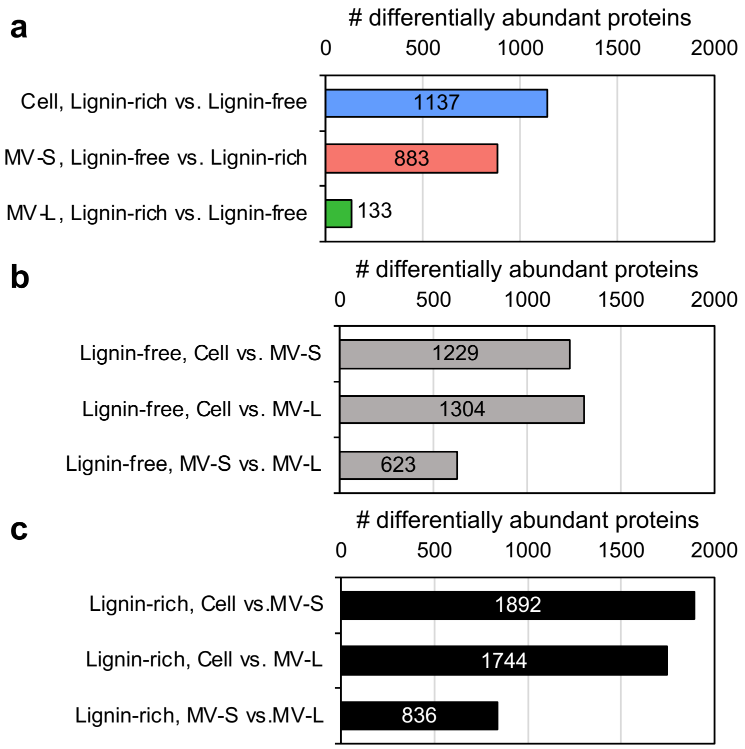

Figure S9. Number of differentially abundant proteins in all comparisons made.
(**a**) In a given compartment across media types. (**b**) In lignin-free cultivations across spatial compartments. (**c**) In lignin-rich cultivations across spatial compartments. Significance was determined using adjusted *p*-values ($p_{adj.}$<0.05 deemed significant).

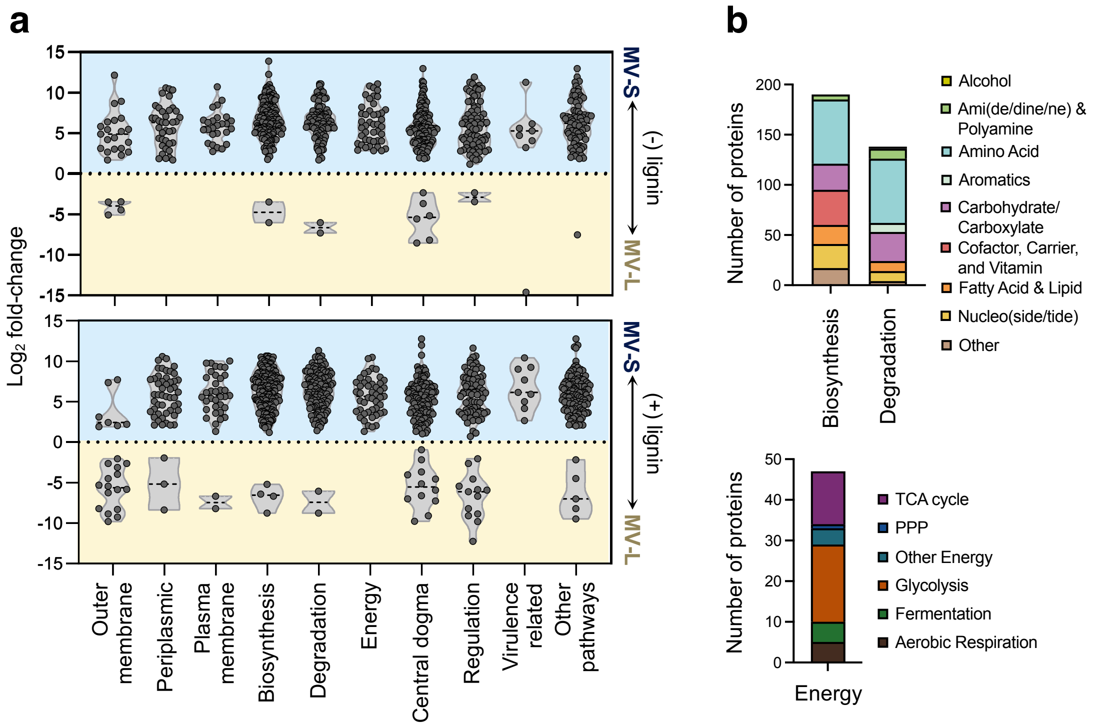

Figure S10. MetaCyc spatial and functional analysis of differentially-abundnat proteins.
(**a**) MetaCyc spatial and functional classification of the significantly differentially-abundant proteins between the MV-S and MV-L in lignin-free and lignin-rich cultivations at 72 h. Each point represents the $\log_2$ fold-change for a protein with a $p_{adj.} < 0.05$ classified in each category. (**b**) Subsystem breakdown for MV-S-enriched proteins in Biosynthesis, Degradation, and Energy pathways from the MetaCyc classification. All analyses performed on data from three biological replicates.

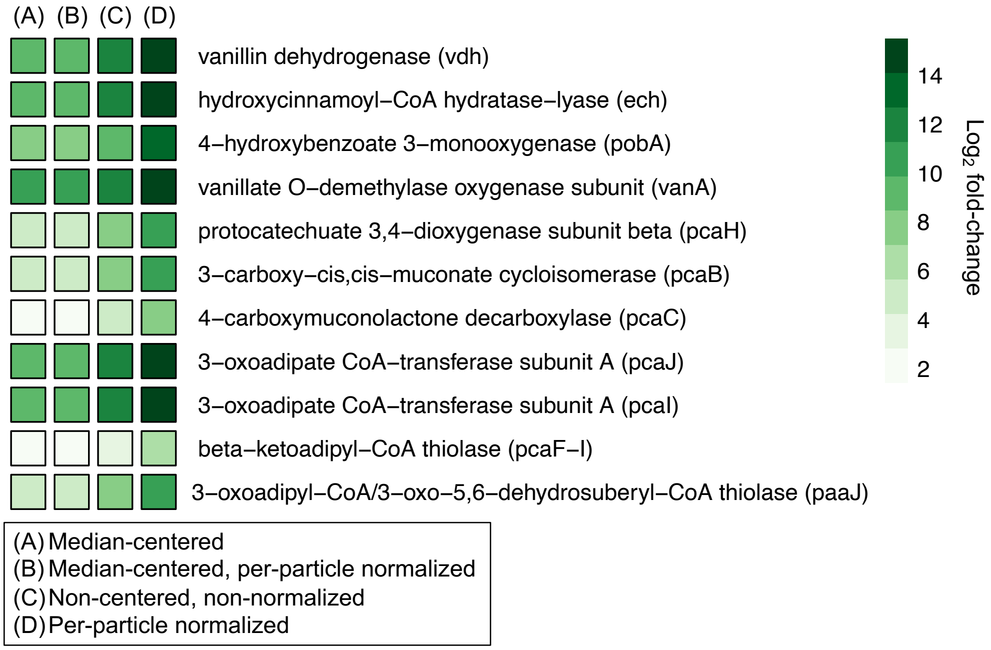

Figure S11. Heatmap comparison of log$_2$ fold-change between MV$_S$ and MV$_L$ in lignin-rich media at 72 h across data centering/normalization strategy.

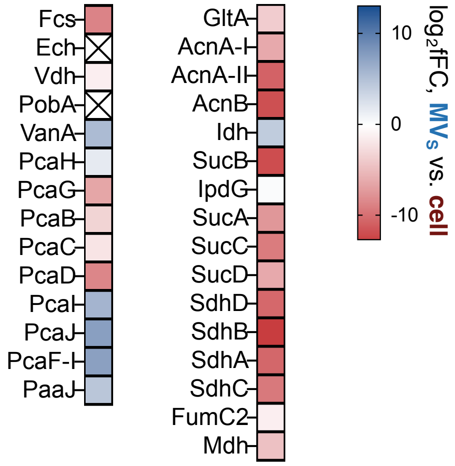

Figure S12. Heat map of differential abundance of beta-ketoadipate and TCA cycle related proteins between the MV_S and cell pellet.

X” indicates $p_{adj.}$>0.05 so fold-change is not plotted.

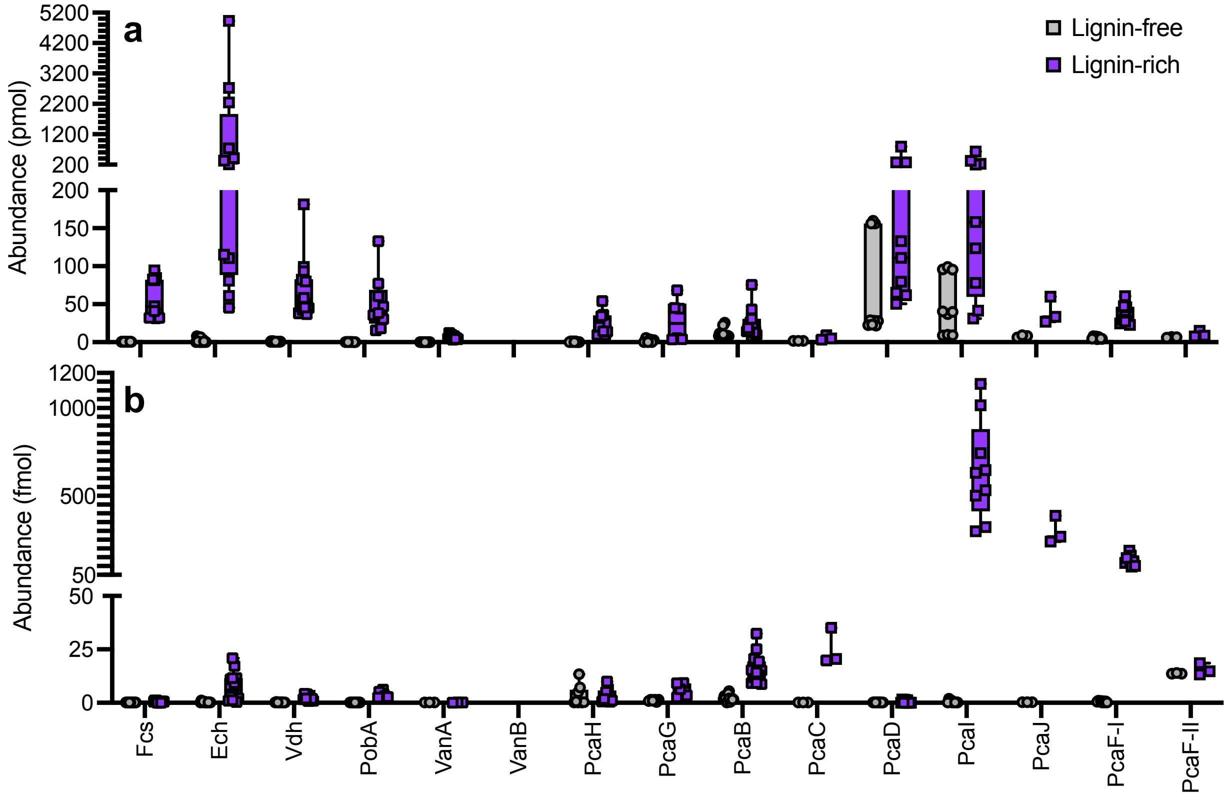

Figure S13. Abundance of each protein as detected by 3-5 unique peptides for (a) cell pellet samples and (b) pooled MV samples for both lignin-free and lignin-rich experiments.
Each individual peptide abundance is shown as a point, and boxes represent the min-to-max values. Cell pellet values are shown as picomole and MV values are shown as fmol.

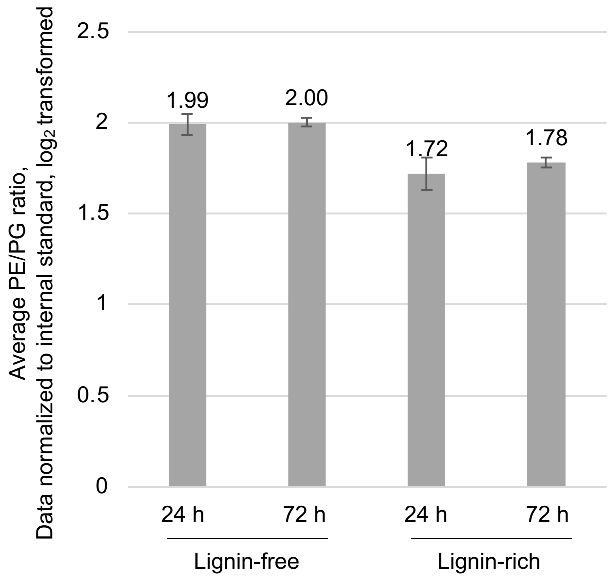

ANOVA Comparison	P-value	F	SumSq
**Lignin**	0.000061	58	0.18
**Time**	0.28	1.3	0.0042
**Lignin:Time Interaction**	0.46	0.61	0.0019

Figure S14. Phosphatidylethanolamine (PE) to phosphatidylglycerol (PG) ratios in cell pellets. Data were normalized and log2 transformed. Results from a two-way ANOVA are shown in the table.

SUPPLEMENTAL TABLES

Table S1. AF4 collection times.

Sample name	Condition	Hour	Replicate	Fraction	Fraction Range- No focusing (min)
A1-24	Lignin-free	24	1	1	1-15
				2	15-43
A2-24	Lignin-free	24	2	1	1-19
				2	19-43
A3-24	Lignin-free	24	3	1	1-19
				2	19-43
A1-72	Lignin-free	72	1	1	1-20
				2	20-43
A2-72	Lignin-free	72	2	1	1-20
				2	20-43
A3-72	Lignin-free	72	3	1	1-27
				2	27-43
B1-24	Lignin-rich	24	1	1	1-14
				2	14-51
B2-24	Lignin-rich	24	2	1	1-14
				2	14-51
B3-24	Lignin-rich	24	3	1	1-14
				2	14-48
B1-72	Lignin-rich	72	1	1	1-13.5
				2	13.5-48
B2-72	Lignin-rich	72	2	1	1-13.5
				2	13.5-47
B3-72	Lignin-rich	72	3	1	1-13
				2	13 – 50.5

Table S2. Particle counts.

Fraction	Sample name	Condition	Replicate	Hour harvested	Count per sample	Count per mL
Fraction 1	A1-24	Lignin-free	1	24	5.87E+09	4.19E+08
Fraction 1	A2-24	Lignin-free	2	24	7.92E+09	4.40E+08
Fraction 1	A3-24	Lignin-free	3	24	8.69E+09	4.83E+08
Fraction 1	A1-72	Lignin-free	1	72	7.57E+09	3.98E+08
Fraction 1	A2-72	Lignin-free	2	72	1.56E+10	8.22E+08
Fraction 1	A3-72	Lignin-free	3	72	1.55E+10	5.97E+08
Fraction 2	A1-24	Lignin-free	1	24	2.52E+08	9.01E+06
Fraction 2	A2-24	Lignin-free	2	24	2.98E+07	1.24E+06
Fraction 2	A3-24	Lignin-free	3	24	3.07E+07	1.28E+06
Fraction 2	A1-72	Lignin-free	1	72	5.35E+07	2.33E+06
Fraction 2	A2-72	Lignin-free	2	72	6.34E+07	2.76E+06
Fraction 2	A3-72	Lignin-free	3	72	1.74E+07	1.09E+06
Fraction 1	B1-24	Lignin-rich	1	24	6.06E+08	4.66E+07
Fraction 1	B2-24	Lignin-rich	2	24	4.42E+09	3.40E+08
Fraction 1	B3-24	Lignin-rich	3	24	9.44E+08	7.26E+07
Fraction 1	B1-72	Lignin-rich	1	72	6.24E+08	4.99E+07
Fraction 1	B2-72	Lignin-rich	2	72	9.71E+08	7.77E+07
Fraction 1	B3-72	Lignin-rich	3	72	1.08E+09	8.98E+07
Fraction 2	B1-24*	Lignin-rich	1	24	1.48E+09	8.00E+07
Fraction 2	B2-24	Lignin-rich	2	24	4.41E+09	1.19E+08
Fraction 2	B3-24	Lignin-rich	3	24	4.22E+09	1.24E+08
Fraction 2	B1-72	Lignin-rich	1	72	5.65E+09	1.64E+08
Fraction 2	B2-72	Lignin-rich	2	72	6.27E+09	1.87E+08
Fraction 2	B3-72	Lignin-rich	3	72	7.20E+09	1.92E+08

*Particle counts represent values from one AF4-MALS technical replicate; all others are technical duplicates.

Table S3. Particle sizes.

Fraction	Condition	Replicate	Hour harvested	Average Radius (nm)	Error (nm)	Radius Range (nm)
1	Lignin-free	1	24	53	6	36-67
1	Lignin-free	2	24	47	11	21-65
1	Lignin-free	3	24	52	13	20-69
1	Lignin-free	1	72	59	14	30-88
1	Lignin-free	2	72	58	14	32-87
1	Lignin-free	3	72	46	14	17-68
2	Lignin-free	1	24	88	19	67 -150
2	Lignin-free	2	24	114	15	68-142
2	Lignin-free	3	24	98	16	71-147
2	Lignin-free	1	72	108	13	90-162
2	Lignin-free	2	72	101	12	87-160
2	Lignin-free	3	72	101	9	77-129
1	Lignin-rich	1	24	46	11	16-61
1	Lignin-rich	2	24	53	7	25-62
1	Lignin-rich	3	24	54	12	21-78
1	Lignin-rich	1	72	50	8	21-64
1	Lignin-rich	2	72	50	8	24-62
1	Lignin-rich	3	72	49	9	29-62
2	Lignin-rich	1	24	126	22	80-167
2	Lignin-rich	2	24	146	28	87-198
2	Lignin-rich	3	24	140	31	82-202
2	Lignin-rich	1	72	150	34	92-219
2	Lignin-rich	2	72	147	32	81-218
2	Lignin-rich	3	72	135	22	85-176

Table S4. Rank-based overlap (rbo) values.

Comparison	Rbo, all proteins	Rbo, top 100 proteins
Lignin-rich 72 h, Small vs. Large MVs	0.584	0.040
Lignin-rich 72 h, Small MV vs. Cell	0.517	0.060
Lignin-rich 72 h, Large MV vs. Cell	0.457	0.020
Lignin-free 72 h, Small vs. Large MVs	0.649	0.000
Lignin-free 72 h, Small MV vs. Cell	0.575	0.000
Lignin-free 72 h, Large MV vs. Cell	0.551	0.000
Small MV 72 h, Lignin-free vs. Lignin-rich	0.606	0.040
Large MV 72 h, Lignin-free vs. Lignin-rich	0.559	0.240
Cell 72 h, Lignin-free vs. Lignin-rich	0.683	0.040

Table S5. Log$_2$ fold-changes for beta-ketoadipate pathway enzymes.
Shown for the following comparisons: MV-S vs. cell at 24 h, MV-S vs. cell at 72 h, MV-S vs. MV-L at 72 h. Comparisons of MV-L at 24 h was not conducted due to the poor sample quality. n/s indicates the result from the differential abundance test indicated the abundance was not statistically significant.

	Lignin-rich cultivations						
	MV-S vs. Cell, 24 h		MV-S vs. Cell, 72 h		MV-S vs. MV-L, 72h		
**Locus**	**log2 fold-change**	**adjusted p-value**	**log2 fold-change**	**adjusted p-value**	**log2 fold-change**	**adjusted p-value**	**Annotation**
PP_3356	-8.63	2.97E-06	-8.63	2.97E-06	n/s	n/s	feruloyl-CoA-synthetase (fcs)
PP_3357	-1.20	2.74E-03	-1.20	2.74E-03	8.99	5.86E-07	vanillin dehydrogenase (vdh)
PP_3358	n/s	n/s	n/s	n/s	9.36	6.25E-06	hydroxycinnamoyl-CoA hydratase-lyase (ech)
PP_3537	n/s	n/s	n/s	n/s	7.11	1.45E-06	4-hydroxybenzoate 3-monooxygenase (pobA)
PP_3736	5.44	2.46E-07	5.44	2.46E-07	9.89	4.09E-07	vanillate O-demethylase oxygenase subunit (vanA)
PP_3737	n/s	n/s	n/s	n/s	n/s	n/s	
PP_4656	1.68	1.52E-03	1.68	1.52E-03	5.02	3.23E-06	protocatechuate 3,4-dioxygenase subunit beta (pcaH)
PP_4655	-6.41	3.64E-05	-6.41	3.64E-05	n/s	n/s	protocatechuate 3,4-dioxygenase subunit alpha (pcaG)
PP_1379	-3.22	9.74E-06	-3.22	9.74E-06	5.21	1.24E-05	3-carboxy-cis,cis-muconate cycloisomerase (pcaB)
PP_1381	-2.02	7.18E-03	-2.02	7.18E-03	2.70	6.69E-04	4-carboxymuconolactone decarboxylase (pcaC)
PP_1380	-8.49	6.63E-07	-8.49	6.63E-07	n/s	n/s	3-oxoadipate enol-lactonase (pcaD)
PP_3952	7.48	3.53E-06	7.48	3.53E-06	9.10	6.04E-07	3-oxoadipate CoA-transferase subunit A (pcaJ)
PP_3951	5.92	1.54E-06	5.92	1.54E-06	9.33	2.66E-07	3-oxoadipate CoA-transferase subunit A (pcaI)
PP_1377	7.39	1.61E-07	7.39	1.61E-07	1.42	3.30E-04	beta-ketoadipyl-CoA thiolase (pcaF-I)
PP_3280	4.59	1.07E-05	4.59	1.07E-05	4.87	7.58E-06	3-oxoadipyl-CoA/3-oxo-5,6-dehydrosuberyl-CoA thiolase (paaJ)

Table S6. Linear model analysis of untargeted lipidomics data from MVs.
Linear model was built using the “lm” function in R. All factors were treated as categorial. The effect size in the scale of the different metrics and represents the average difference between experimental factors. The effect size confidence interval (CI) shows the 1% and 99% bounds respectively in the parentheses.

**Quantitative Metric**	**Experimental factor**	**Effect size (98% CI)**	**p-value**
PE/PG Ratio	Media (Free → Rich)	-9.5 (-12, -6.6)	4.9e-8
PE/PG Ratio	Time (24hr → 72hr)	-0.71 (-3.5, 2.1)	0.54
PE/PG Ratio	Size (Small → Large)	-3.1 (-6.0, -0.3)	0.01
Average FA Length	Media (Free → Rich)	0.033 (0.011, 0.055)	0.0012
Average FA Length	Time (24hr → 72hr)	0.0036 (-0.018, 0.025)	0.69
Average FA Length	Size (Small → Large)	-0.015 (-0.034, 0.006)	0.090
Average FA Unsaturation	Media (Free → Rich)	0.099 (-0.002, 0.20)	0.022
Average FA Unsaturation	Time (24hr → 72hr)	-0.048 (-0.15, 0.053)	0.25
Average FA Unsaturation	Size (Small → Large)	0.084 (-0.017, 0.18)	0.048

Table S7. Linear model analysis of untargeted lipidomics data from cell pellets.
Linear model was built using the "lm" function in R. All factors were treated as categorial. The effect size in the scale of the different metrics and represents the average difference between experimental factors. The effect size confidence interval (CI) shows the 1% and 99% bounds respectively in the parentheses.

Quantitative Metric	Experimental factor	Effect size (98% CI)	p-value
PE/PG Ratio	Media (Free → Rich)	-0.62 (-0.84, -0.41)	1.9e-5
PE/PG Ratio	Time (24hr → 72hr)	0.09 (-0.13, 0.30)	0.29
Average FA Length	Media (Free → Rich)	0.023 (0.009, 0.035)	8.2e-4
Average FA Length	Time (24hr → 72hr)	0.10 (0.09, 0.12)	3.3e-9
Average FA Unsaturation	Media (Free → Rich)	0.013 (0.005, 0.020)	9.3e-4
Average FA Unsaturation	Time (24hr → 72hr)	-0.011 (-0.018, -0.003)	0.0031
